# Structure–Spectroscopy Correlations for Intermediate Q of Soluble Methane Monooxygenase: Insights from QM/MM Calculations

**DOI:** 10.1021/jacs.1c01180

**Published:** 2021-04-22

**Authors:** Christine E. Schulz, Rebeca G. Castillo, Dimitrios A. Pantazis, Serena DeBeer, Frank Neese

**Affiliations:** † Max-Planck-Institut für Kohlenforschung, Kaiser-Wilhelm-Platz 1, 45470 Mülheim an der Ruhr, Germany; ‡ Max Planck Institute for Chemical Energy Conversion, Stiftstr. 34-36, 45470 Mülheim an der Ruhr, Germany

## Abstract

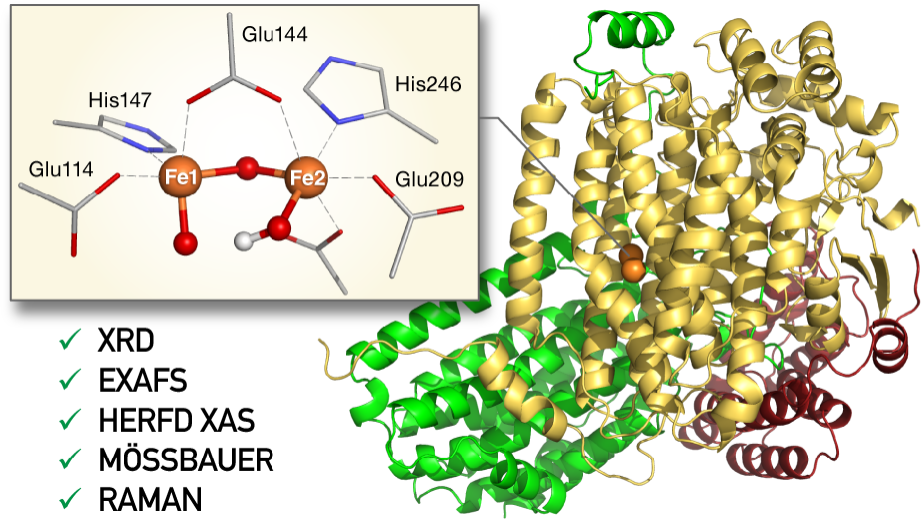

The determination of the diiron core intermediate structures involved in the catalytic cycle of soluble methane monooxygenase (sMMO), the enzyme that selectively catalyzes the conversion of methane to methanol, has been a subject of intense interest within the bioinorganic scientific community. Particularly, the specific geometry and electronic structure of the intermediate that precedes methane binding, known as intermediate Q (or MMOH_Q_), has been debated for over 30 years. Some reported studies support a bis-μ-oxo-bridged Fe(IV)_2_O_2_ closed-core conformation Fe(IV)_2_O_2_ core, whereas others favor an open-core geometry, with a longer Fe–Fe distance. The lack of consensus calls for a thorough re-examination and reinterpretation of the spectroscopic data available on the MMOH_Q_ intermediate. Herein, we report extensive simulations based on a hybrid quantum mechanics/molecular mechanics approach (QM/MM) approach that takes into account the complete enzyme to explore possible conformations for intermediates MMOH_ox_ and MMOH_Q_ of the sMMOH catalytic cycle. High-level quantum chemical approaches are used to correlate specific structural motifs with geometric parameters for comparison with crystallographic and EXAFS data, as well as with spectroscopic data from Mössbauer spectroscopy, Fe K-edge high-energy resolution X-ray absorption spectroscopy (HERFD XAS), and resonance Raman ^16^O–^18^O difference spectroscopy. The results provide strong support for an open-core-type configuration in MMOH_Q_, with the most likely topology involving mono-oxo-bridged Fe ions and alternate terminal Fe-oxo and Fe-hydroxo groups that interact via intramolecular hydrogen bonding. The implications of an open-core intermediate Q on the reaction mechanism of sMMO are discussed.

## 1 Introduction

**Figure 1:**
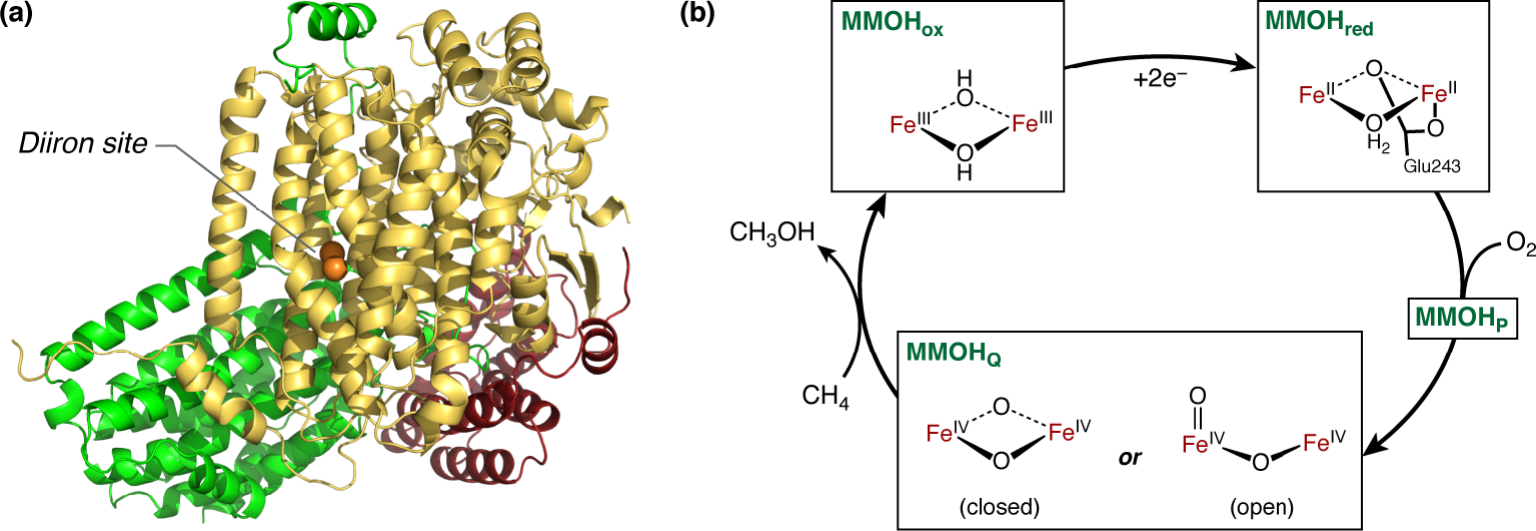
(a) Structural overview of the MMOH protein, and its three subunits (α, yellow; β, green; γ, red), with the α subunit harboring the diiron site. (b) Abbreviated reaction cycle of MMOH, featuring the resting state MMOH_ox_, MMOH_red_, and the MMOH_Q_ intermediate. Note that for MMOH_ox_ the protonation states of the μ-oxo bridges are not unambiguously resolved.

In nature, methane is oxidized to methanol under ambient temperature and pressure by methane monooxygenases (MMOs) in methanotrophic bacteria via O_2_ activation.^1−3^ These enzymes can be categorized into two main classes: particulate MMO (pMMO) that contains a copper active site and the iron-containing soluble MMO (sMMO), which is expressed in the absence of copper.^4−9^ The sMMO enzyme consists of four protein units: the hydroxylase (MMOH), which contains the diiron active site, a regulatory protein (MMOB), which facilitates substrate access to the active site, and a reductase (MMOR) and the auxiliary protein MMOD, which is thought to inhibit MMOH.^10,11^ MMOH itself consists of an αβγ motif as shown in Figure 1. The diiron active site is located close to the MMOH surface, to which it is connected by a channel, leading to a cavity at the active site.^12,13^ The active site in MMOH contains two iron ions bridged by glutamate and solvent-derived oxygen ligands.^14^ In the diferric resting state (MMOH_ox_),^15^ the two high-spin irons are antiferromagnetically coupled, leading to a spin singlet ground state. Upon two-electron reduction provided by NADH-reduced MMOR, the diferrous state (MMOH_red_) is able to react with dioxygen, yielding a peroxo intermediate (MMOH_p_). After breaking of the O–O bond, the intermediate MMOH_Q_ is formed. This is the key intermediate in the catalytic cycle that carries out methane activation and has thus been the subject of extensive experimental and theoretical studies.^8,14,16−28^

MMOH_Q_ was first observed in 1993 by UV–vis absorption spectroscopy.^25^ Mössbauer spectroscopy revealed that it consists of two antiferromagnetically coupled Fe(IV) ions.^26^ Although there is no crystal structure of MMOH_Q_, the extended X-ray absorption fine structure (EXAFS) data were interpreted in terms of an unusually short Fe–Fe distance of 2.46 Å in connection with two short Fe–O bonds to each iron.^22^ Resonance Raman experiments, in conjunction with isotope labeled oxygen, showed that both oxygen atoms of the O_2_ molecule are incorporated in MMOH_Q_.^17^ Combining this information led to the notion that MMOH_Q_ contains a closed bis-μ-oxo (“diamond”) Fe_2_O_2_ core (Figure 1b). Although MMOH_Q_ has inspired numerous synthetic model complexes,^29−32^ the proposed geometry with a short Fe–Fe distance has not been reproduced by any synthetic models.^20,32,33^ Recent high energy resolution fluorescence detected (HERFD) EXAFS studies of MMOH_Q_ revisited the older assignments and corrected the Fe–Fe distance to a much longer separation of ∼3.4 Å,^28^ which supports other geometries, commonly referred to as open-core conformations (Figure 1b). Further, even if indirect, support for open cores comes from synthetic model complexes, which are more active toward C–H bond activation than those containing closed Fe_2_O_2_ cores.^34^ Moreover, Fe K-edge HERFD-XAS studies showed that the pre-edge region in MMOH_Q_ does not match with the pre-edge of the Fe(IV)Fe(IV) close core model complex, but it is rather similar to the open-core model complex pre-edges.^27^ In the same study, TDDFT calculations were used to rationalize the XAS spectra, providing confidence to the assignments and to the methodological approach.

The new experimental results on MMOH_Q_ urge for a careful and thorough revision of past spectroscopic interpretations, and the development of new structural models that can accommodate the current data. This can be achieved through the development of computational models that correctly connect structural features of MMOH_Q_ with the currently known spectroscopic observables. Computational studies of this system have a long history in assisting experimental interpretation and suggesting a possible mechanism.^19,21,23,35−69^ Aside from structure predictions,^19,36,37,40,41,60^ both the transition from MMOH_P_ to MMOH_Q_^21,38,39,42,43,45,52,68^ and the substrate oxidation^35,38,44,46−51,53,54,56−59,61−65,67,69^ have been studied. However, most studies have been typically limited by the lack of extensive connections to spectroscopic data, in part because this information was not available at the time they were conducted. These limitations include the use of very small quantum mechanical (QM) cluster models, without including a realistic ligand set and without consideration of protein-derived structural constraints in early computational studies. In contrast, studies that attempted to incorporate the effect of the environment in a quantum mechanics/molecular mechanics approach (QM/MM) concentrated on intermediates in dioxygen^21^ or methane^67^ activation or comparison to other diiron enzymes.^57^

Importantly, it appears that past computational investigations of the nature of MMOH_Q_ and of possible reaction mechanisms have been largely based on models that are no longer consistent with the most up-to-date experimental data. The incomplete account of the protein environment in most past studies, as well as the wealth of new information on the electronic and geometric structure of MMOH_Q_, necessitates a thorough reappraisal of both the modeling approach and the structure–spectroscopy correlations themselves. Herein, we revisit the questions on the structure and properties of MMOH_Q_ from the ground up based on a QM/MM approach that is ensured to be converged with respect to the effect of the protein and with emphasis on utilizing the available sources of spectroscopic data (including EXAFS, Mössbauer spectroscopy, X-ray absorption, and resonance Raman spectroscopies) in the theoretical evaluation of new models. First, we study the resting state MMOH_ox_ and then we focus on a wide variety of models to describe MMOH_Q_, examining how their geometric features and protonation states correlate with the complete range of spectroscopic properties currently available on the system. Our results enable us to narrow down the possible formulations of MMOH_Q_ and to propose revised schemes for the conceivable reaction mechanisms of sMMO.

## 2 Models and Methods

### 2.1 Structural Analysis of XRD Models

**Figure 2:**
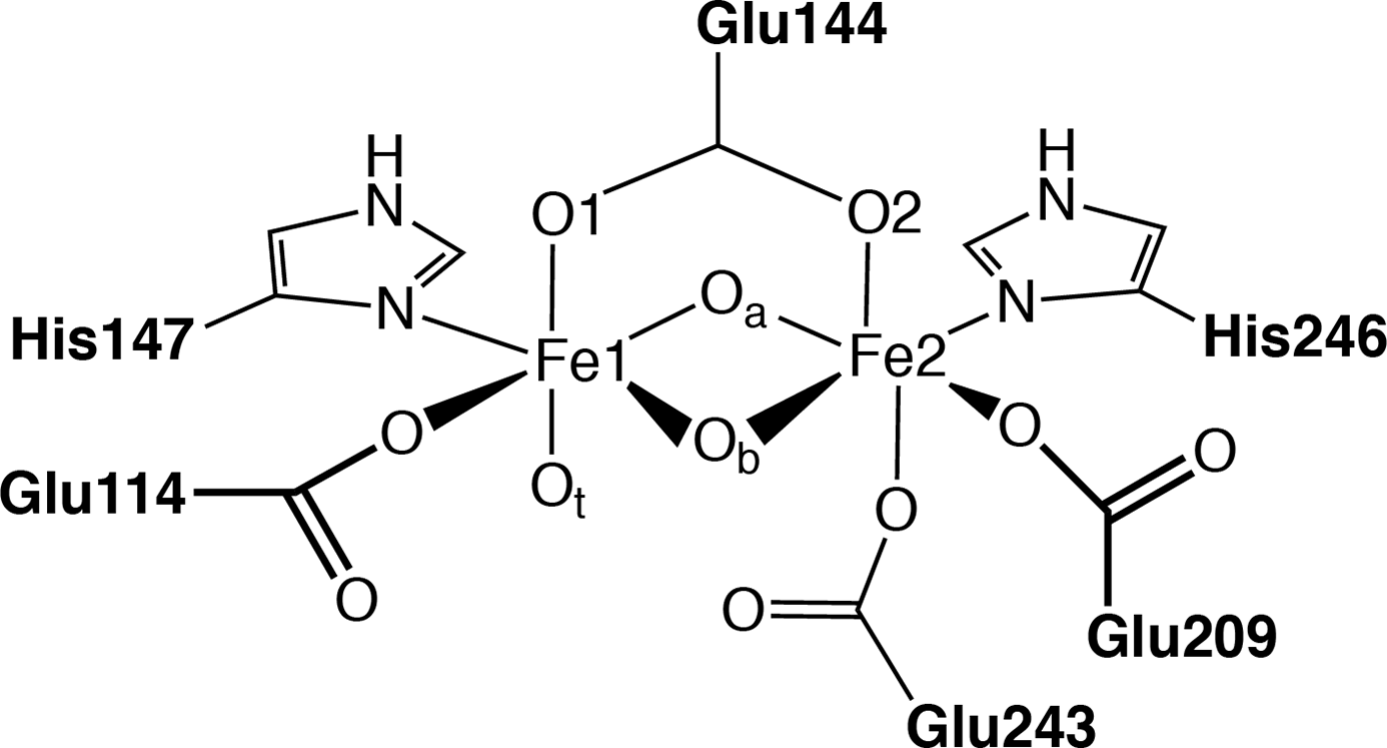
Schematic representation of the diironactive site including the first ligand sphere: O_t_ denotes the terminal oxygen, O_a_ is pointing toward the histidines, and O_b_ is directed toward the water-containing cavity that leads to the surface of the protein. In MMOH_ox_, the protonation states for these oxygens are unknown.

The first step in building a computational model of the MMOH diiron active site (Figure 2) is an analysis of the existing structural data from crystallography. Structural information about MMOH_ox_ can be obtained using XRD or EXAFS Spectroscopy.^2,14,22,28,70^ In total, 28 individual structures of MMOH are available in the RCSB.^12,14,71−74^ Yet, all of these were obtained under different conditions because the maturation of MMOH was studied using various apo-structures.^72^ Structures of the maturated MMOH are available in both resting and reduced states: MMOH_ox_^14,71^ and MMOH_red_.^73^ Starting from these, large studies were done on pH dependence^73^ or the effect of alcohols or alcoholates as bridging ligands to the irons.^74^ Most recently, a structure of the MMOH+MMOB complex was resolved.^13,75^ However, not all of these crystal structures (Table S1) contain an intact Fe_2_O_2_ unit. For this reason, we have performed a careful analysis and selection of crystallographic data, as described in the Supporting Information and presented in Table S2.

**Figure 3:**
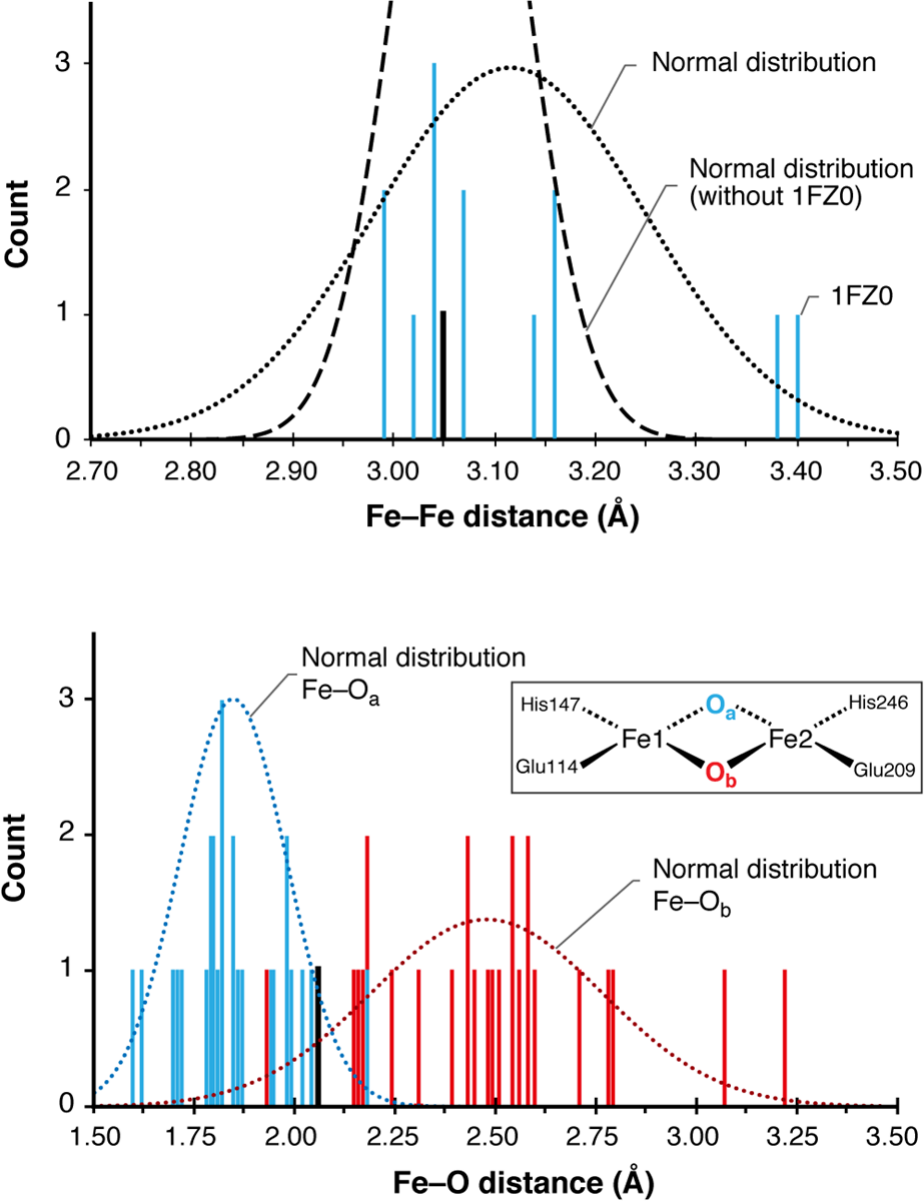
Analysis of Fe–Fe and Fe–O distances of nine intact MMOH_ox_ active sites. Distance count rounded to two digits. The HERFD-EXAFS scatter for the Fe–Fe distance and short Fe–O distance is shown in black.

The results of this analysis with respect to the distribution of Fe–Fe and Fe–O distances in presumably intact XRD models are presented in Figure 3 and discussed in the Supporting Information. If mixed-valence outliers (1FZ0^73^) are excluded, we would reach an average Fe–Fe distance of 3.07 ± 0.06 Å, which is in excellent agreement with the HERFD-EXAFS distances of 3.06 Å^28^ and previous EXAFS.^76^ The mean Fe–O_a_ distance is 1.85 ± 0.13 Å, and the mean Fe–O_b_ distance is 2.41 ± 0.22 Å. Figure 3 suggests that O_a_ is more constrained than O_b_, which adopts a wider variety of bonding positions, presumably as a result of its environment (O_b_ is oriented toward a water filled cavity). An important question is the compatibility between XRD and HERFD-EXAFS results. EXAFS only resolves average distances between Fe and its surrounding atoms, being typically unable to distinguish scatterers with similar atomic weight such as O and N if they are at similar distance. The shortest Fe–O/N distances were fitted to 1.96 Å^77^ or 2.06 Å,^28^ with a resolution of 0.17 Å. Since the short Fe–O distances are expected at ca. 1.85 Å, they will overlap with Fe–O/N distances to the Glu and His ligands. HERFD-EXAFS resolves a longer Fe–O/N distance of 2.48 Å, which can be attributed to either the Fe–O_t_ or the longer Fe–O_b_ distance, in agreement with the XRD averages of 2.48 Å (Fe–O_b_) and 2.31 ± 0.12 Å (Fe–O_t_).

### 2.2 Construction of the QM/MM Model

**Figure 4:**
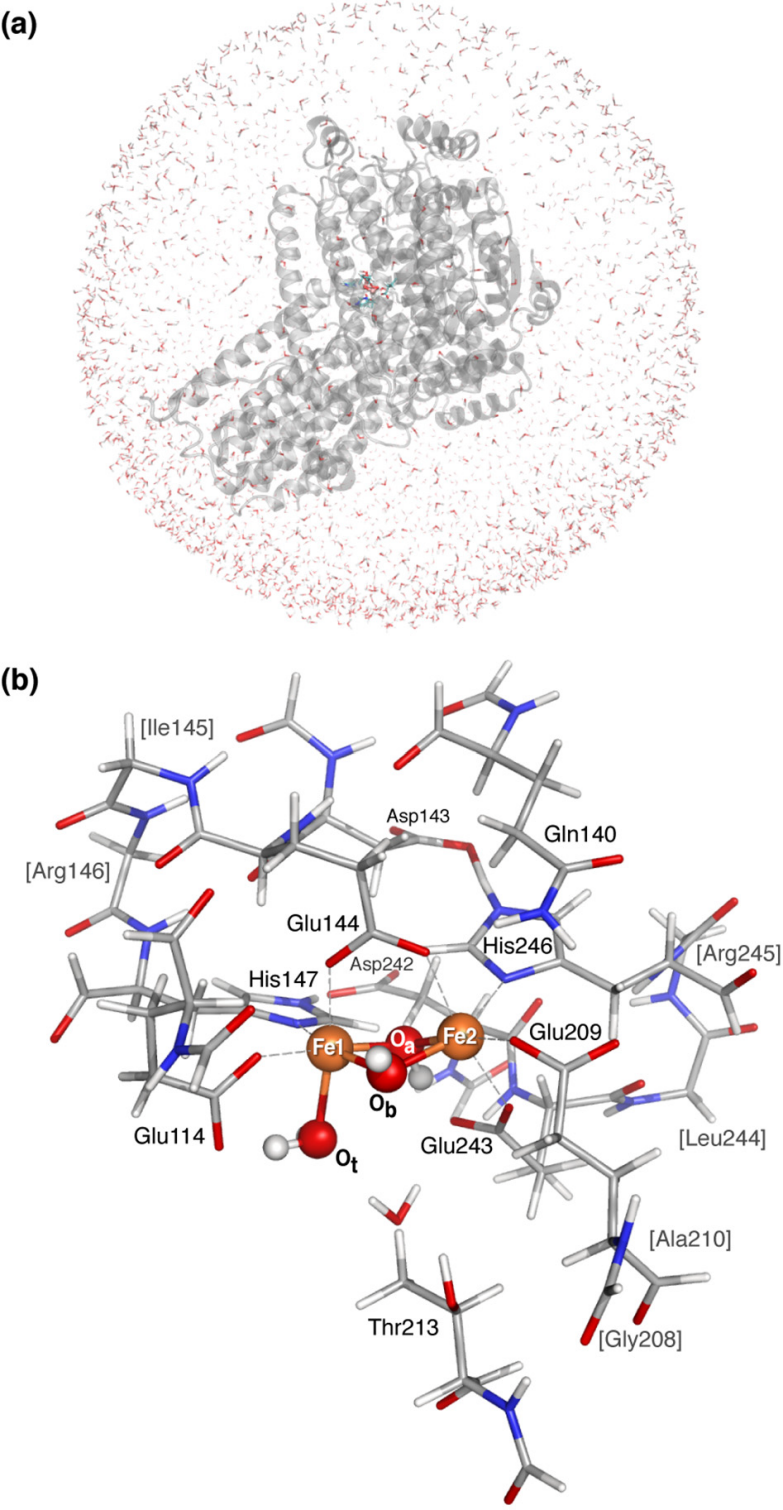
(a) Complete system overview of the solvated MMOH protein, (b) first and second solvation sphere around the diiron active site as used in the QM subsystem, including link atoms.

Based on the crystal structure analysis, the 1MTY^14^ crystal structure was chosen as a starting point for the QM/MM calculations. Hydrogens and water were added using the CHARMM27 force field.^78^ The crystallographic water positions were maintained. In addition, a water droplet with at least 10 Å buffer around the protein was created, leading to a total of 90,000 atoms (Figure 4a). Hydrogen and water positions were optimized for 10,000 steps before a stepwise equilibration for 50,000 steps in total. The QM region consists of the first and second layer of amino acids around the Fe_2_O_*x*_ cluster, in total around 210 atoms depending on the protonation state of the bridging oxo groups (Figure 4b). Since the putative bis-μ-oxo structure of MMOH_Q_ is remotely similar to the core geometry of MMOH_ox_, the same structure was used as a starting point for the geometry optimizations for the MMOH_Q_ models. Only models with bridging Glu243 ligands required more flexibility in the backbone of the glutamate, than the 1MTY structure could accommodate. Hence, here the hydroxylase part of the 4GAM crystal structure^12^ was used as a starting point. Note that due to the limited resolution of 2.9 Å and the bridging Glu243 in the MMOH_ox_ state, the 4GAM structure was not considered a fitting starting point for MMOH_ox_ models. The MM structure was prepared analogous to the 1MTY structure, with a total system size of 90,000 atoms. For the QM models, the analogous residues were chosen, so that the number of QM atoms derived from both crystal structures is the same.

### 2.3 Computational Details

All calculations were performed with the ORCA program package.^79,80^ QM/MM calculations used an interface of ORCA with NAMD.^81−83^ Geometries were optimized in the high-spin state following the same protocol as ref (27) using the BP86 functional,^84,85^ with def2-TZVP basis sets^86^ on Fe, N, and O, while def2-SVP basis sets were used for C and H. The RI-J approximation was used with def2/J basis sets. For the optimizations, TightOpt settings with tighter integration grids (Grid6) were used. Dispersion corrections were included using D3BJ.^87^ Mössbauer parameters were calculated according to ref (88) using the B3LYP functional^89,90^ with core-property CP(PPP)^91^ basis sets. For the Fe atoms, tighter grids and integration accuracy (SpecialGridIntAcc 7) were used. Mössbauer spectra were simulated using the “mf” program written by Dr. E. Bill.^92^ X-ray absorption spectra were calculated using TD-DFT,^93,94^ again with the B3LYP functional with a def2-TZVP basis. Spectra were plotted with an applied broadening of 1.0 eV using the orca_mapspc utility. An energy shift of 150.85 eV was applied, as determined by the difference between calculated XAS spectra and experimental HERFD XAS pre-edge energies for model complexes.^27^ We note that due to the relatively limited HERFD XAS data that are available, a smaller calibration set (Figure S1) was utilized as compared to previous TFY/transmission XAS calibration studies.^95,96^ A graphical summary of the experimental vs calculated energy shift is given in Figure S2. It should be noted that the exact shift depends on the chosen methodology.^96^ Similarly, the scaling factor for the calculated vs experimental areas can be obtained as shown in Figure S3. The scaling factor depends on how exactly the calculated area is determined. Here, the area was determined by calculating the sum of the oscillator strengths. Arguably the quality of the extrapolation increases with the number of data points, therefore the pre-edges were separated into individual peaks. Following this analysis, the pre-edge areas are scaled by a factor of 5.97. Nonresonant Raman spectra were calculated using the same level of theory as the geometry optimizations. Isotope labeled Raman spectra were obtained using the orca_vib program. The Raman spectra were plotted with an applied broadening of 5 cm^–1^ using the orca_plot utility.

## 3 Results and Discussion

### 3.1 Models of MMOH_ox_

**Figure 5:**
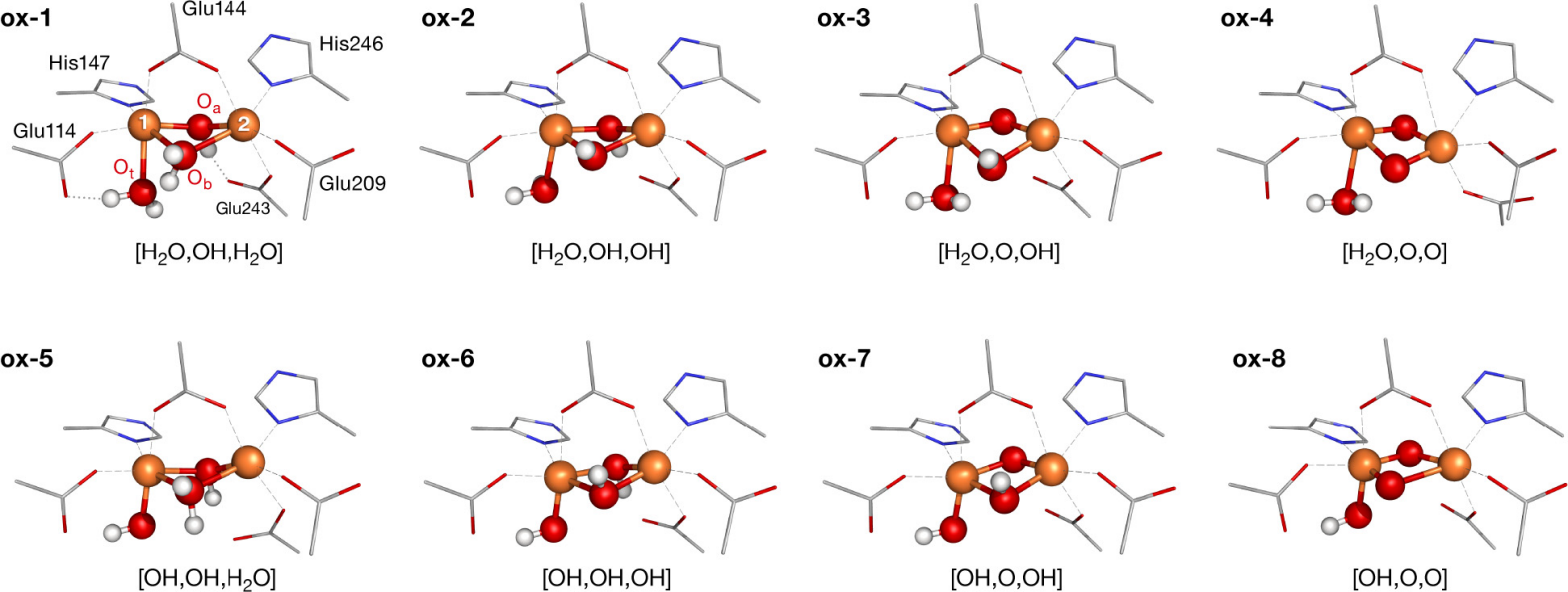
Geometries of the Fe_2_O_*x*_ core from optimized MMOH_ox_ structures. A schematic representation of the MMOH_ox_ core structures is provided in Figure S4.

To directly compare possible protonation states of MMOH_ox_, a set of models (Figure S4) was constructed in order to test the flexibility of the system and the ability of our computational approach to differentiate between different protonation states. These models start with a terminal water and a μ-OH/μ-OH_2_ bridging ligand (model **ox-1**). The bridging ligands are successively deprotonated up until model **ox-4** with a bis-μ-oxo core. Similarly, models with terminal hydroxo ligands were constructed, again with a μ-OH/μ-OH_2_ bridge (model **ox-5**). These were also systematically deprotonated leading up to a bis-μ-oxo core at model **ox-8**. The geometries of the diiron cores after geometry optimization are shown in Figure 5.

#### 3.1.1 Molecular Structure of MMOH_ox_

Some general trends in the models can be deduced directly from Table 1. The models with terminal waters have a longer Fe–O_t_ distance than the models with terminal hydroxo ligands. Additionally, all models have an asymmetric core: the Fe–O_a_ distances are shorter than the Fe–O_b_ distances, even for models with the same protonation state in both bridges. And last, the calculated distances to O_t_ and O_b_ are shorter than the XRD average. A plausible explanation for this is that the terminal oxygen (O_t_) and the frontal bridging oxygen (O_b_) are pointing toward a water filled cavity, and are thus affected by hydrogen bonding to water in this cavity. Since water molecules are not resolved in the crystal structures, it can be assumed that they are not tightly locked in rigid positions. Instead, there are many possible water positions, leading to many possible hydrogen bond confirmations. This is reflected in the variety of bond lengths in the crystal structures, leading to a larger standard deviation.

**Table 1:** Structural Data for the Possible MMOH_ox_ Structures shown in Figure 5^a^

Model	Fe_1_–Fe_2_	Fe_1_–O_t_	Fe_1_–O_a_	Fe_2_–O_a_	Fe_1_–O_b_	Fe_2_–O_b_
**ox-1**	3.29	2.21	1.96	1.97	2.29	2.36
**ox-2**	3.07	2.20	1.97	1.98	2.05	2.17
**ox-3**	2.95	2.25	1.85	1.85	2.19	2.08
**ox-4**	2.67	2.24	1.88	1.83	1.89	1.88
**ox-5**	3.31	1.90	2.10	1.93	2.21	2.33
**ox-6**	3.09	1.84	2.03	1.91	2.22	2.06
**ox-7**	2.92	1.88	1.92	1.78	2.15	2.03
**ox-8**	2.91	1.82	1.74	1.93	1.71	2.33
XRD Average	3.07	2.31	1.80	1.90	2.35	2.61

a All values in Å.

**Figure 6:**
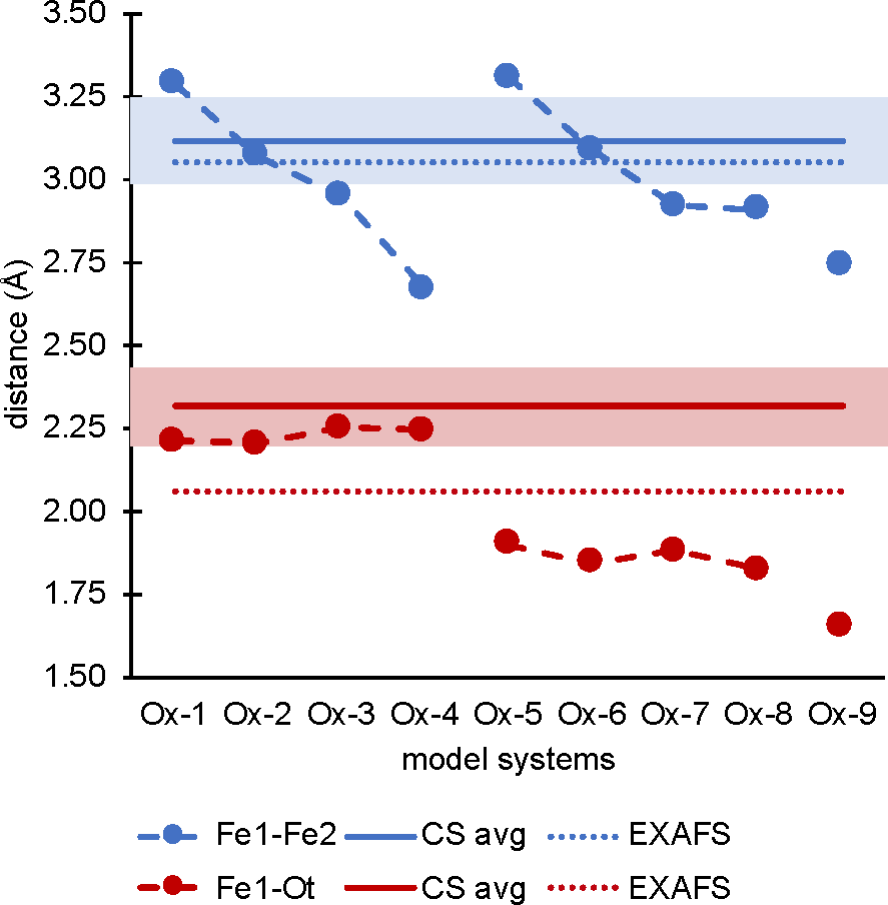
Graphical representation of the geometry analysis. Left: Difference of the Fe–Fe and Fe–O_t_ bond lengths in the models vs the crystal structure average of the 13 intact crystal structures presented in Table 1 and EXAFS distances.^28^ Full lines denote the crystal structure average, dotted lines denote the EXAFS fits, and pastel bars denote the standard deviation. Note that only the shorter EXAFS Fe–O distances were included.

A more detailed graphical comparison of the QM/MM results with the XRD analysis is presented in Figure 6. As a second level of comparison, the EXAFS distances have been included. This figure compares the difference of each distance to the corresponding crystallographic average. In doing so, the models can be ranked according to the number of distances they represent well. Additionally, the standard deviation of the crystal structures is given, to provide a trust range for each distance. Based on this comparison, models with a terminal hydroxo ligand can be excluded, since the Fe–OH distances are too short compared to the crystal structure average. Intermediate protonation states yield the best agreement for the Fe–Fe distances. The graphical comparison for the Fe–O distances is given in Figure S5, and a similar figure with the ligand distances is given in Figure S6. The Fe–O distances are best represented by models that do not feature a large spread of distances. The closest fit is achieved for models with two hydroxo bridges. Therefore, the geometric parameters of models **ox-2** and **ox-3** agree best with the available experimental data.

#### 3.1.2 Electronic Structure of MMOH_ox_

The basis for the property calculations is the electronic structure of MMOH_ox_. Both irons were treated as high spin irons (local *M*_*S*_ = 5/2), coupled antiferromagnetically. This is achieved by first calculating the high-spin state (*M_s_* = 5) and then flipping the spin on one iron-center followed by reconverging the self-consistent field equations to the broken-symmetry solution with *M*_*S*_ = 0. The correct broken-symmetry state is confirmed by inspection of the atomic spin populations and corresponding orbitals. The presence of five mostly metal-centered singly occupied orbitals on each site was confirmed, by inspection of the Mulliken spin populations (Table S3). Antiferromagnetic coupling is observed for all models except model **ox-4**, which is very weakly ferromagnetically coupled. For the MMOH_ox_ models with a terminal water ligand (**ox-1** to **ox-4**), less than 5% of the spin population is found on the oxygen bridges, independent of protonation state of the bridging ligand. For models with a terminal hydroxo ligand (**ox-5** to **ox-8**), the spin population on the bridging oxygens has increased to 10–25%, and has significantly increased on the terminal oxygen (∼17–38%), which in some cases may be considered to develop partial radical character. However, there is no clear correlation of these values to the exchange coupling constant. As a representative example, the corresponding orbitals and overlap integrals of model **ox-3** are shown in Figure S7.

#### 3.1.3 Mössbauer Parameters of MMOH_ox_

In the next step, models can be evaluated using their spectroscopic properties, beginning with the Mössbauer parameters. The calculation of Mössbauer parameters focuses on the isomer shift, which is proportional to the charge density at the nucleus, and the quadrupole splitting, derived from the electric field gradient at the iron nucleus.^91^ After proper calibration of the isomer shift,^88^ the predicted isomer shifts tend to be more reliable than the calculated quadrupole splittings.^97,98^ Thus, for more complicated iron systems, the analysis usually focuses on the isomer shift.^99^ An overview on the calculated and experimental Mössbauer parameter for MMOH_ox_ is given in Table 2. The experimental Mössbauer data show two ferric irons with almost identical isomer shifts.^15^ The quadrupole splitting shows pH dependence.^100^ Simulations of the experimental parameters and calculated Mössbauer are presented in Figures S8 and S9.

**Table 2:** Calculated Mössbauer Parameters for the Models Presented in Figure 5, Compared to Experimental Mössbauer Data for the MMOH_ox_ Main Component^a^

	Δ*E*_q_		δ	
Model	Fe_1_	Fe_2_	Fe_1_	Fe_2_
**ox-1**	–1.67	–0.95	0.49	0.44
**ox-2**	–1.64	0.87	0.51	0.47
**ox-3**	–2.09	–1.54	0.51	0.50
**ox-4**	2.07	1.38	0.49	0.51
**ox-5**	–1.52	–1.18	0.49	0.45
**ox-6**	–1.79	–1.32	0.50	0.48
**ox-7**	–2.51	–1.44	0.51	0.48
**ox-8**	3.10	–0.98	0.78	0.17
Experiment				
ref (15)	1.20	0.95	0.50	0.50
ref (100), pH 7	1.16	0.87	0.51	0.50
ref (100), pH 9	1.77	0.70	0.54	0.50

a Note that the iron labels for the experimental data are arbitrary. All values in mm/s.

An additional level of complexity arises from the fact that there are two ways of generating the MMOH_ox_ intermediate: since it is the resting state, it can either be utilized as isolated^15,100^ or generated by oxidizing MMOH_red_, as done in recent studies.^28^ For the latter, an additional signal in the Mössbauer spectrum is observed.^101−103^ One hypothesis states that these originate from antisymmetric exchange that has been observed in model complexes.^101^ In single turnover studies two species for the MMOH_ox_ state were found.^102^ They referred to the second as MMOH_ox_(sl), for the slow reacting species, which is thought to be closer to the MMOH_red_ geometry.

The isomer shift of the calculated ox models are all of the same order of magnitude, with the exception of **ox-8**. Several models are within or close to the experimental difference of the two isomer shifts (Δmaxδ 0.4). The quadrupole splitting, as expected, is more sensitive and shows greater fluctuation in the different models. The simulation of the calculated Mössbauer parameters is given in Figure S9. The doublet type spectrum, as observed in earlier studies or at lower pH, is reproduced by model **ox-5**. Several models show a doublet with split tips, resulting from two irons with similar, yet distinct splittings. In models **ox-2** and **ox-7** the quadrupole splittings of the two irons are so different that two doublets are resolved. While this is reminiscent of the second species found in MMOH_ox_ powder spectra,^28,101^ it should be noted that the splitting of **ox-7** is larger than experimentally observed. The protonation state of the bridging oxygens might give information on the geometry of MMOH_ox_(sl), but clearly further experimental investigation is needed. From the Mössbauer calculations, model **ox-8** can be safely excluded from the list of possible MMOH_ox_ models. Yet, since all other models are rather close in the isomer shift, a definitive distinction based on these data alone are not possible. However, both **ox-2** and **ox-3**, which were the best models based on the geometric structure analysis, also lead to calculated Mössbauer parameters that are consistent with experiment.

#### 3.1.4 XAS Pre-edges of MMOH_ox_

**Figure 7:**
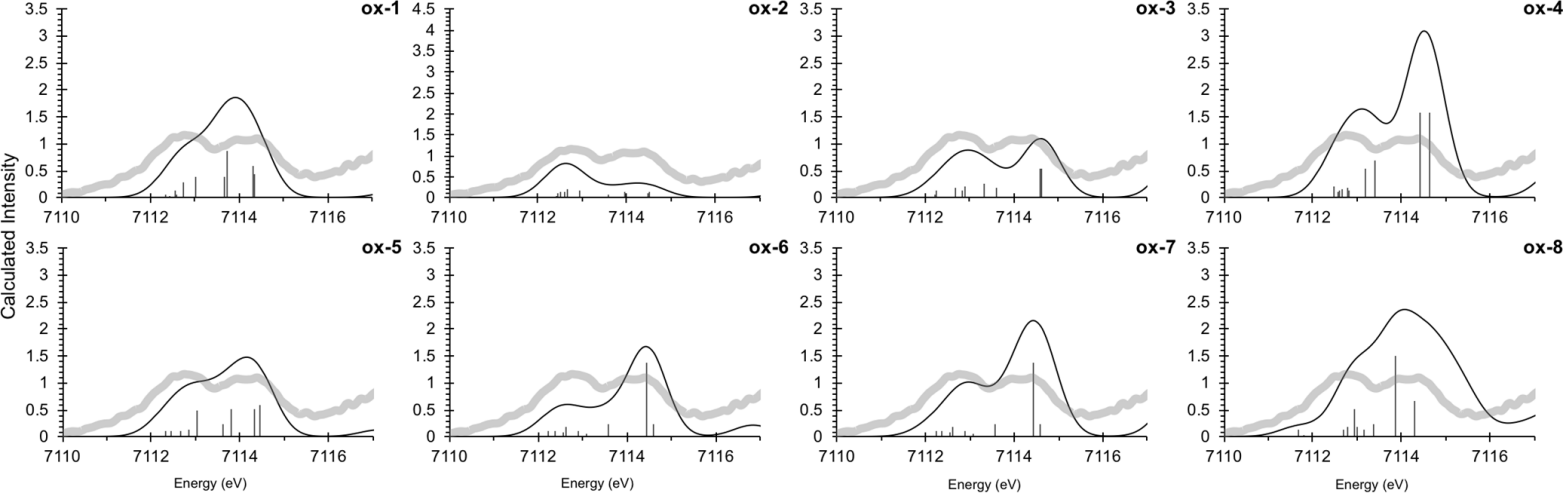
Calculated XAS pre-edge spectra for selected MMOH_ox_ models (black) on top of the experimental spectrum (gray).^27^

X-ray absorption spectroscopy offers an additional way of evaluating the electronic structure of these models. Within a molecular orbital picture, the XAS pre-edge features correspond to transitions from the 1s metal orbital to the unoccupied 3d orbitals.^27^ This leads to 10 transitions for a diferric di-iron site, leading to two peaks which, in a simple picture, can be understood as resulting from the splitting between the *t*_2*g*_ and *e*_*g*_ manifold of the pseudo-octahedral metal sites. The exact nature of the main transitions that give rise to this pattern have been thoroughly discussed before.^27^ In model complexes,^27^ it was found that equal protonation states on the μ-O bridging ligands yield a symmetric spectrum. The transitions to the *t*_2*g*_-set (*d*_*xy*_, *d*_*xz*_, and *d*_*yz*_, orbitals) comprised in the lower energy pre-edge peak show a lower intensity than the high energy pre-edge peak. The transitions overlap perfectly, indicating orbital degeneracy due to a high symmetry at the iron cores. For model complexes with asymmetric bridging ligands (μ-oxo and μ-hydroxo), the transitions do not overlap since the irons are unequal. The *d*_*z*^2^_ orbitals were found to be the highest in energy and intensity, followed by the *d*_*x*^2^ – *y*^2^_ orbitals. The *t*_2*g*_-set was found grouped together at lower energies, leading to a second lower energy pre-edge feature. The experimental XAS pre-edge shows two signals for MMOH_ox_, separated by ∼2 eV. Looking the calculated pre-edges for the protein (Figure 7), lower symmetry is expected, due to the nature of the diiron site and ligands involved. Hence, complete degeneracy would not be expected even for symmetrically protonated bridging ligands

A quantitative comparison with experiment is given in Table 3. Most models show an IWAE which is slightly larger than experiment, with the exception of **ox-2**, which is shifted to slightly lower energy. This is largely a result of the differences in the pre-edge intensity distributions. Most models reproduce the experimental shape given by two peaks, with the second calculated feature having higher intensity than the first one. Only model **ox-2** and **ox-8** deviate from this. In the case of model **ox-2**, the second pre-edge peak (∼7114.4 eV) is lower in both energy and intensity; hence, the IWAE is shifted toward lower energies. Looking at the pre-edge areas, model **ox-3** is closest to the experimental area of 13.37 units, and certainly within the estimated ∼10% error due to the limited data for the area regression. This is followed by **ox-6** and **ox-5**, whereas other models deviate more strongly.

**Table 3:** Difference between Calculated and Experimental IWAE and XAS Pre-edge Areas of the MMOH_ox_ Models

model	**ox-1**	**ox-2**	**ox-3**	**ox-4**	**ox-5**	**ox-6**	**ox-7**	**ox-8**	Exp^27^
IWAE (eV)	7113.63	7113.18	7113.71	7113.91	7113.63	7113.87	7113.87	7113.8	7113.3
Area	19.62	8.03	14.28	31.97	17.78	15.95	22.85	28.20	13.37

#### 3.1.5 Summary on MMOH_ox_ Models

By combining the results of geometries and spectroscopy, useful conclusions regarding the structure of the MMOH_ox_ intermediate can be reached. Models featuring a terminal water ligand and bridging hydroxo ligands agree best with the crystal structures. From Mössbauer spectroscopy, it can be seen that the two irons are expected to have a very similar local environment; this is achieved for **ox-5**, which features a terminal hydroxo ligand with a bridging hydroxo and water ligand. Among models with terminal water ligands, **ox-3** and **ox-4** show the best agreement, although differences among the two irons are observed. Models **ox-2** and **ox-7** will not be considered further, because they do not agree with the low-pH Mössbauer experiment. The XAS pre-edges show that although the shape and width of the calculated pre-edges are relatively consistent among models, changes in the protonation state of the bridging and terminal ligands lead to subtle differences in the distribution of intensities. The best agreement in terms of spectral shape, IWAE, and area is observed for model **ox-3**. Therefore, on average, we suggest that model **ox-3** may be considered the best candidate for MMOH_ox_.

### 3.2 Models for MMOH_Q_

**Figure 8:**
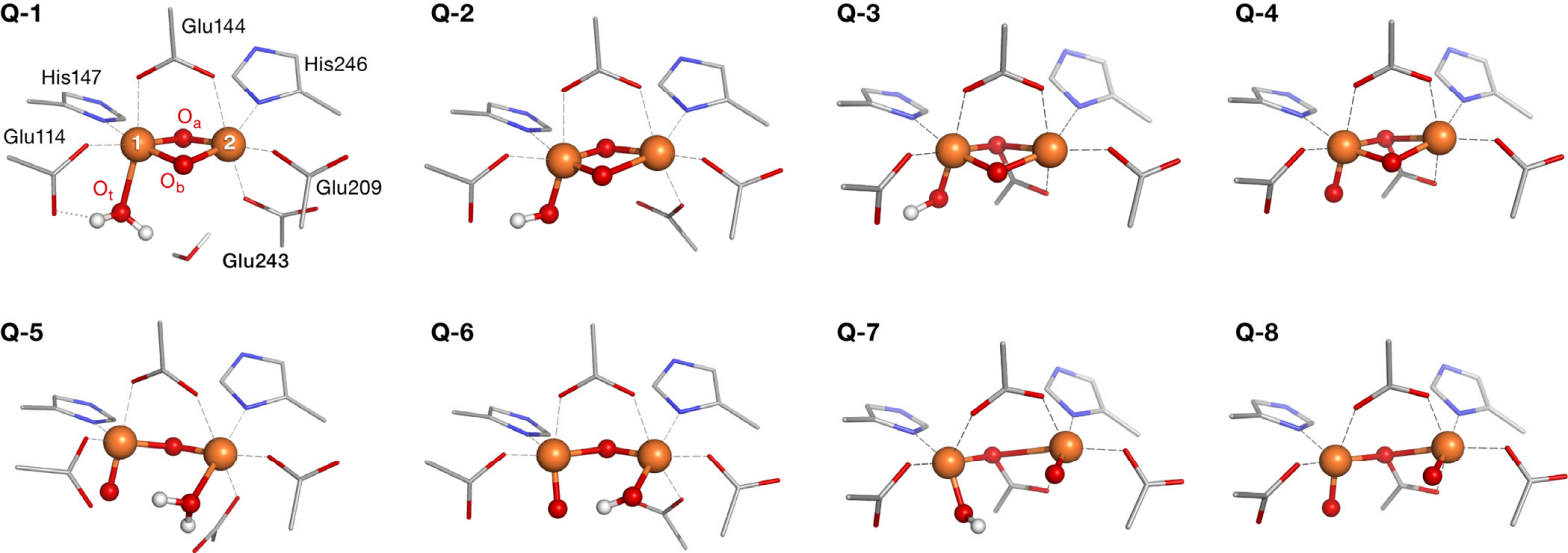
Optimized structures of the suggested core motifs for MMOH_Q_. A schematic representation of the cores presented in Figure S10.

Based on previous studies supporting closed- and open-core conformations for the key intermediate in sMMO, a set of models was constructed for the MMOH_Q_ active site. The selection of plausible models include the following: (1) bis-μ-oxo bridged cores; (2) μ-OεGlu243 bridged diamond cores, inspired from the MMOH_red_ structure, as suggested recently;^28^ (3) μ-oxo bridged open cores; and (4) μ-OεGlu243 bridged open cores. A schematic representation of all models is shown in Figure S10, while Figure 8 depicts the optimized cores of eight MMOH_Q_ models (**Q-1** to **Q-8**) chosen for further analysis. Among those models, the first four are closed cores, while the last four open up toward the cavity. While the schematic view (Figure S10) allows an easy way of classifying the models, in reality each model is only one member of a larger family of conceivable structures that differ in subtle details concerning the orientation of hydrogen bonds and the arrangement of surrounding waters. In the real system, at room temperature, there most certainly is some structure fluctuation that interconverts members of the same structural family. However, a detailed analysis of these dynamic processes is outside of the scope of this paper.

#### 3.2.1 Molecular Structure of MMOH_Q_

From the data presented in Table 4, it is evident that the Fe–Fe distances clearly distinguish the closed- and open-core models, with the closed cores having Fe–Fe distances between 2.8 and 3.0 Å and the open cores having distances larger than 3.2 Å. We note that these distances are all longer than the originally reported EXAFS distance of 2.46 Å,^22^ but agree quite well with the respective closed- and open-core molecular models.^27^ For both the closed- and open-core models the presence of the μ_1_-bridging Glu243 (**Q-3**, **Q-4**, **Q-7**, and **Q-8**) results in an elongation of the Fe–Fe distance by ∼0.1 Å for the closed-core models, and more than 0.4 Å for the open-core models. Models **Q-7** and **Q-8** show an even larger Fe–Fe distance, than the HERFD EXAFS distance for the Fe–Fe vector of ∼3.4 Å.^28^ Therefore, the best agreement with the experiment would be an open-core model with slightly less interaction than observed in the μ-oxo bridging models **Q-5** and **Q-6**.

**Table 4:** Central Distances for the Central Iron Unit of QM/MM Optimized MMOH_Q_ Models in Figure 8

model	Fe_1_–Fe_2_	Fe_1_–O_a_	Fe_2_–O_a_	Fe_1_–O_b_	Fe_2_–O_b_	Fe_2_–O_t_	Fe_1_–O_t_
**Q-1**	2.80	1.84	1.86	1.84	1.80	–	2.25
**Q-2**	2.84	1.72	2.12	1.74	1.94	–	1.78
**Q-3**	2.98	1.96	2.40	1.86	1.86	–	1.70
**Q-4**	3.03	2.04	2.34	1.83	1.90	–	1.64
**Q-5**	3.20	1.85	1.73	–	–	2.19	1.65
**Q-6**	3.21	1.72	1.86	–	–	1.66	1.80
**Q-7**	3.85	1.95	2.75	–	–	1.65	1.72
**Q-8**	3.68	1.98	2.38	–	–	1.64	1.64

The Fe–O distances within the core are more scattered than in the MMOH_ox_ models. In contrast to the Fe–Fe distances, no clear pattern is observed. However, within the MMOH_Q_ models, the Fe–O distances can be nicely grouped depending on the protonation state. Fe–O_t_ distances to terminal oxo ligands are the shortest, between 1.64 and 1.68 Å (**Q-4** to **Q-8**). Distances to terminal hydroxo ligands are found to be 1.78 Å for closed-core models (**Q-1** and **Q-3**) and 1.79 to 1.82 Å for open-core models (**Q-6** and **Q-7**).

**Figure 9:**
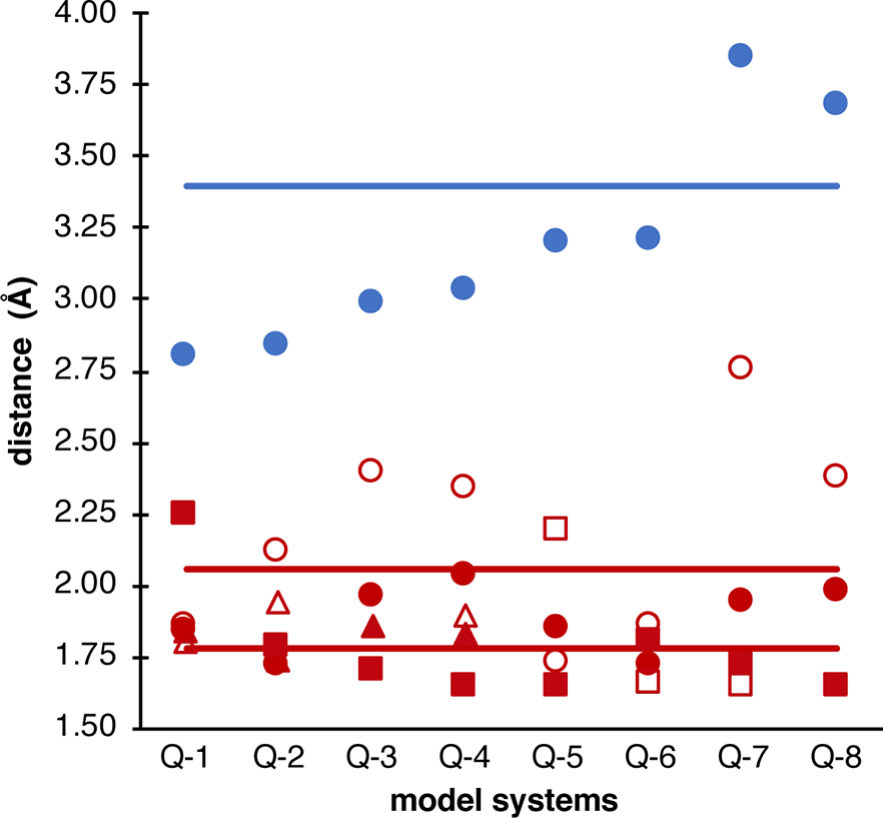
Graphical representation of the geometry analysis: Difference of the Fe–Fe (blue) and Fe–O bond lengths in the MMOH_Q_ models vs the EXAFS distance. Full lines denote the EXAFS distances, with the Fe–Fe distance being estimated at 3.4 Å. The Fe–O distances are separated into Fe1 (filled) and Fe2 (empty), and O_a_ (circles), O_b_ (triangles), and O_t_ (squares).

MMOH_Q_ models can also be analyzed by the asymmetry of their cores. The Fe−μ-O distances in models **Q-5** and **Q-6** show an interesting pattern of asymmetry, with one short Fe−μ-O distance of 1.73 Å and a longer distance at 1.86 Å. An even more asymmetric pattern is observed in the bis-μ-oxo model **Q-2**, with a short Fe–O bond of 1.72/1.74 Å and a longer bond of 1.94/2.12 Å. In contrast, the Fe−μ-oxo distances in model **Q-1** are very symmetric, with distances between 1.80 and 1.86 Å. The longest Fe–O distances are observed for the Fe–OεGlu243 distances, in both closed- (**Q-3** and **Q-4**) and open-core models (**Q-7** and **Q-8**). Compared to the experiment (Figure 9), the averaging of the core distances in the respective model becomes important. For the sake of simplicity, the ligand distances are separately shown in Figure S11. Several models show that the core distances can be nicely averaged in a way that is consistent with the two EXAFS distances 1.78 and 2.06 Å.^28^ Only model **Q-6** lacks the longer Fe–O distances in the core. However, the Fe–O/N distances to the glutamate and histidine ligands also contribute to this longer EXAFS distance. As shown in Figure S11, these agree well with the EXAFS distances.^28^

In conclusion, the geometric analysis shows that open-core models agree with the EXAFS data, while the closed cores all exhibit Fe–Fe distances that are too short. However, the Oε-Glu243 bridged open-core models **Q-7** and **Q-8** are overestimating relative to experiment. Hence the best agreement is observed for oxo bridged open-core models, like **Q-5** and **Q-6**.

#### 3.2.2 Electronic Structure of MMOH_Q_

The MMOH_Q_ intermediate consists of two high-spin Fe(IV) ions which are antiferromagnetically coupled. Hence, all models were set up initially as locally high spin (*M*_*S*_ = 2 assumed for each Fe ion; i.e., four unpaired electrons) but in a broken symmetry state with total *M*_*S*_ = 0. The results were analyzed by inspection of the atomic spin populations and of the corresponding orbitals. The corresponding orbital transformation offers a convenient way of analyzing broken-symmetry wave functions by identifying the nonorthogonal valence-bond-like magnetic orbital pairs in antiferromagnetically coupled systems.^104^ The procedure transforms the spin-unrestricted canonical orbitals into a set that diagonalizes their overlap matrix, i.e. each spin-up orbital has nonzero overlap with at most one spin-down orbital. The resulting orbitals are ordered according to their overlap integrals into a group of essentially doubly occupied spin-up/spin-down orbital pairs (spatial overlap close to unity), a group of unmatched magnetic orbitals where the spin-up and spin-down counterparts are localized on different sites of the molecule (spatial overlap between zero and one), and finally a set of unmatched spin-up orbitals in case the number of α electrons exceeds the number of β electrons. For a broken-symmetry *M*_*S*_ = 0 state of a dimer consisting of two high-spin Fe(IV) we expect four pairs of corresponding orbitals (see Figure S12 for two representative examples). These were indeed found for all models, with the exception of **Q-4**. These pairs of corresponding orbitals fall into categories of stronger coupling (overlap range of 0.7–0.8) and weaker coupling (overlap range of 0.1–0.2). In general, deviations may arise when the calculations converge to *M*_*S*_ = 0 solutions with locally intermediate-spin iron ions or with significant ligand radical character. The Mulliken spin populations for all models are provided in Tables S5 and S6. We observe that different models may have a different degree of spin localization, often deviating from the formal integer values expected for high-spin d^4^ iron ions. Additionally, some of the models, including **Q-2** and particularly **Q-4**, have noticeable asymmetry in the two Fe ions in terms of local spin populations.

As shown in Table S5, a substantial amount of spin is found on several oxygen ligands. Especially the terminal oxo ligands show significant spin population. This is independently found in closed- (**Q-4**) and open-core models (**Q-5**–**Q-8**). The largest spin population is found on the terminal oxo ligand of **Q-4** resulting from strong spin delocalization over the Fe–O_t_ moiety, conferring high radical character to O_t_. For the glutamate bridged models (**Q-7**, **Q-8**), almost no spin is found on the oxygen of the bridging glutamate (O_a_). For μ-oxo bridged open cores (**Q-5**, **Q-6**), substantial spin is found on the bridging oxygen (O_a_). An interesting comparison is between **Q-1** and **Q-2**, where in **Q-1** the spin is mostly localized on the irons, while in **Q-2** there is significant spin along the μ-oxo bridges. Inspection of the molecular orbitals confirms that in all cases the HOMO of the system has significant *d*_*z*^2^_ character. Its orientation is determined by the strongest interacting σ ligand. In the case of the diiron site, the μ-oxo ligands, the glutamates, and terminal ligands (H_2_O, OH, oxo) are competing. For irons with a terminal oxo ligand, the *d*_*z*^2^_ orbital on that iron is oriented along the Fe–O_t_ bond. In the closed-core models, the *d*_*z*^2^_ orbitals of the two irons are parallel, with the *xy* plane in the same plane as the μ-oxo bonds. This means that the *d*_*z*^2^_ orbital is oriented along the Fe–OGlu bonds to Glu114. For the open-core models, the orientation of the *d*_*z*^2^_ is determined by the orientation of the terminal oxo moiety. On the other iron, the *d*_*z*^2^_ orbital is oriented along the Fe−μO bond, since this is a stronger σ ligand than the terminal H_2_O or OH ligand.

Again, the QM/MM computational models can be compared to synthetic model complexes.^27,31,105^ The Fe–Fe distances in the QM/MM models and the synthetic complexes agree well. The closed-core model complex and the closed-core computation models (**Q-1**, **Q-2**) show the best agreement. In the open-core models, differences are observed. In the synthetic complexes, all oxygen based ligands lie in the same plane, regardless of whether the complex is labeled as “open” or “closed”. This is not the case in the open-core models in the protein and may have important implications for the comparability of the electronic structure and spectroscopic properties between the two. Here the Fe-Ot unit is perpendicular to the Fe−μO–Fe plane. The reason for this difference is the different ligand environment, from the rigid chelate ligand in the synthetic complex to the flexible oxygen-rich environment of the protein, embedded in a network of various hydrogen-bonding possibilities. As a consequence, the electronic structure and spectroscopy are not expected to be directly comparable between the QM/MM models of the enzyme and the small synthetic model complexes. Here one has to be cautious, because the model complexes feature iron ions in an intermediate spin state (locally *M*_*S*_ = 1) and their total spin differs from the protein:^27^ the closed-core model is *M*_*S*_ = 0, while the open-core model is *M*_*S*_ = 2. Therefore, no synthetic complex has a direct analogy to the natural system in terms of the electronic structure configuration of the Fe ions. In the following we focus on spectroscopic properties of the QM/MM models developed here.

#### 3.2.3 Mössbauer Parameters of MMOH_Q_

**Figure 10:**
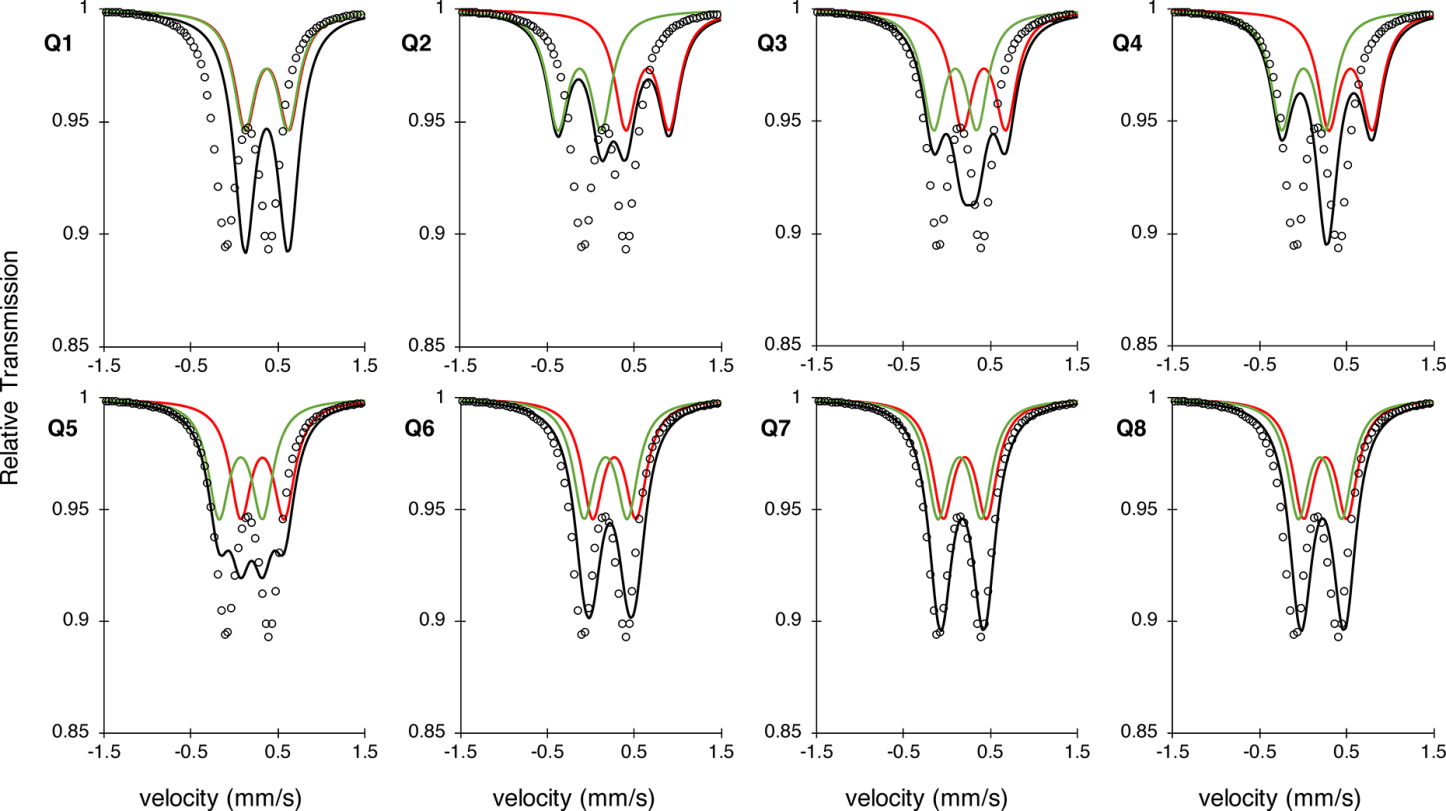
Simulated Mössbauer spectra of the two calculated isotope shifts (red, green) and their resulting spectrum (black), using the average of the experimental quadrupole splitting (0.5). The simulated spectrum using the experimental MMOH_Q_ values (dots) is added to provide a visual comparison that allows judgment of which model represents the experiment most accurately.

The Mössbauer parameters for each model are given in Table 5. As MMOH_Q_ is a reactive intermediate, it cannot be trapped in a pure form and the raid freeze quenched sample utilized for Mössbauer experiments is comprised of at least three species (MMOH_red_, MMOH_ox_, and MMOH_Q_), each with two individual irons. In modeling the experimental Mössbauer spectrum, the MMOH_Q_ component was fit with only one parameter set for two irons, indicating that the two irons in MMOH_Q_ can be considered equal within the resolution of obtained spectra. In the calculations, however, no symmetry constraints were used, leading to inequalities in the two Fe sites. This raises the question, by how much the parameters for each iron can differ, to still result in a two line Mössbauer spectrum, as opposed to a four line spectrum that would be obtained for highly inequivalent irons. To answer this question, the calculated spectra were simulated. Since the determination of quadrupole splitting using DFT is less reliable than the isomer shift,^106^ the comparison here focuses on the isomer shift and the average of the experimental quadrupole splitting were used in the simulation (see Figure S13). The resulting simulations were compared to the simulations using the experimental parameter set (Figure 10).

**Table 5:** Calculated Mössbauer Parameters for the QM/MM Models Presented in Figure 9^a^

	Δ*E*_q_		δ	
	Fe_1_	Fe_2_	Fe_1_	Fe_2_
**Q-1**	0.93	–0.78	0.38	0.37
**Q-2**	2.45	–0.58	0.65	–0.13
**Q-3**	–1.83	1.54	0.43	0.10
**Q-4**	2.22	–2.22	0.54	0.00
**Q-5**	–1.54	0.47	0.32	0.07
**Q-6**	–1.16	–2.53	0.27	0.17
**Q-7**	–0.84	1.31	0.21	0.15
**Q-8**	0.52	1.14	0.25	0.19
ref (27)	0.42		0.15	
ref (28)	0.56		0.18	

a All values in mm/s.

The calculated Mössbauer parameters for models **Q-2**, **Q-3**, **Q-4**, and **Q-5** yield a multiline spectrum, due to the difference in the isomer shifts. This obviously differs from the experimental Mössbauer, which suggests two similar irons. Very good agreement with the experimental observation is found for models **Q-6**, **Q-7**, and **Q-8**. **Q-1** is the most reasonable of any of the closed-core models, however, the calculated isomer shift is too high and outside the typical error expected for such calculations (ca. 0.1 mm/s). Further, **Q-1** has already been eliminated based on the Fe–Fe distance being much too short. This leaves **Q-6**, **Q-7**, and **Q-8** as the most viable candidates for the structure of intermediate Q after the geometric and Mössbauer analysis.

#### 3.2.4 XAS Pre-edge Spectra for MMOH_Q_

Similar to the MMOH_ox_ XAS spectra, the MMOH_Q_ spectra are quite complex due to the ligand set and protein environment. However, in MMOH_ox_, due to the two d^5^ irons only excitations of β-electrons from the core to the valence shell are allowed. By contrast, the Fe(IV) d^4^ configuration in MMO-Q features empty d-based molecular orbitals that can be reached by excitation from either a spin-up or a spin-down electron. Consequently, in MMOH_Q_ for the high spin *S* = 1 Fe(IV) configuration, significant spin polarization is expected to split the pre-edge into α/β *d*_*z*^2^_ components.^96,107^ As discussed elsewhere, this is a crude approximation to the true multiplet splittings that occur by exciting core electrons into empty orbitals in an open-shell system.^107^ The treatment of spin-polarization in TDDFT is considered a “poor man’s approximation” to the multiplet splitting,^96,107^ as discussed multiple times.^27,108^ A splitting is also observed for Fe(IV) *S* = 1 molecular model complexes (∼0.6 eV).^27^ Here caution has to be applied due to the differences in the local spin at the iron. Hence, first these model complex studies^27^ are recalled in detail. It was found that pre-edge shapes and intensities directly reflect the similarity of the local electronic structure of the two metal sites. In closed-core models, the local geometries of the ions are very similar. Consequently, the transitions into the respective *d* orbitals of either iron site are very similar in energy, leading to six different energy transitions. If the two irons show differences in their local symmetry, the inequivalence of the metals lead to 12 different energy transitions. This is the case for the open-core model complexes, as well the protein models. Additionally, it was established in the model complexes that a lower local symmetry in the iron coordination geometry leads to an increase in the pre-edge intensity, as the p–d mixing allows for larger dipole contribution in the pre-edge, which is otherwise dominated by quadrupole contributions for more symmetric complexes.

**Figure 11:**
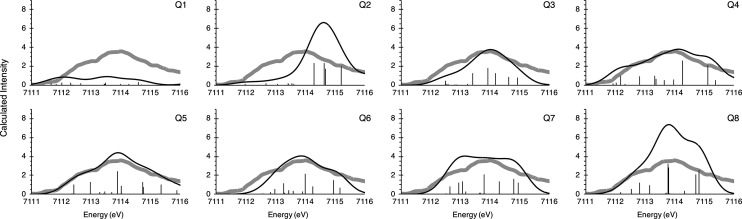
Calculated XAS pre-edge spectra for the MMOH_Q_ models (black) on top of the experimental spectrum (gray).^27^

The experimental XAS pre-edge shows an ∼4 eV wide signal of high intensity, which consists of two grouped transitions, consistent with a distorted local octahedral *O*_*h*_ geometry at each iron. Several models show a similar shape (see Figure 11); however, clearly models **Q-5** and **Q-6** have the best visual agreement, in terms of both the energy and intensity distribution. To assess this more quantitatively, the calculated area, as well as the IWAE, is compared to the experiment in Table 6.

**Table 6:** Difference between Calculated and Experimental XAS Pre-edge Areas and IWAE of the MMOH_Q_ Models

model	**Q-1**	**Q-2**	**Q-3**	**Q-4**	**Q-5**	**Q-6**	**Q-7**	**Q-8**	Exp^27^
IWAE (eV)	7112.8	7114.5	7113.9	7113.9	7113.7	7114.0	7113.8	7113.9	7114
Area	15.32	61.29	41.47	66.28	61.16	46.17	62.57	86.17	42.10

Looking at the calculated IWAEs, most models agree very well with experiment. For the areas, as discussed before, an error of at least 10% is expected. The best agreement is observed for model **Q-3**; however, the IWAE is far too low. **Q-6** is only marginally above the experimental intensity and, importantly, also in good agreement with the IWAE.

The comparison between similar models leads to the unexpected finding of a high intensity pre-edge for the closed-core model **Q-2**. As shown in Table 6, the pre-edge of **Q-2** is as intense as the signal of **Q-5**, without any terminal oxo ligands. The reason for the gain in intensity from model **Q-1** to model **Q-2** is the greater geometric distortion around the irons, and in particular the short Fe–O bonds at the Fe1 site, which provide a mechanism for enhanced p–d mixing via metal–ligand covalency.^109^ A direct comparison is shown in Figure S14. The bond arrangement around the Fe1 of **Q-2** features much shorter ligand bonds, than in the Fe1 of **Q-1**. In summary, the best agreement with the XAS pre-edge experiment is observed for model **Q-6**, in terms of IWAE and pre-edge area. This ties well with the Mössbauer spectroscopy and the geometry analysis presented above.

In the following, the underlying nature of the XAS pre-edge transitions will be analyzed. Note that for the high spin Fe^IV^, with a d^4^ configuration and local spin state of 2, a total of six transitions per iron are expected, one α and five β transitions. Due to the increased asymmetry in the protein compared to the model complexes, for both diamond and closed cores, the two irons are sufficiently inequivalent such that all 12 transitions are found at different energies in the calculated pre-edge spectrum. An assignment for closed-core model **Q-1** and open-core model **Q-6** is presented in Figure S15. The transitions and the resulting orbital picture for the closed-core model **Q-1** and the open-core model **Q-6** are shown in Figure S15 (right panel).

At the lower energy side of the pre-edge of **Q-1**, transitions into the t_2g_ set are found. The signals from the two irons are overlapping and separated from the transitions into the e_g_ set. Next, in increasing energy, are the transitions into the *d*_*z*^2^_ orbitals of each iron, with the transition into the Fe1 *d*_*z*^2^_ orbital at lower energy. The transitions at the highest energy in the pre-edge region are excitations into the unoccupied *d*_*x*^2^ – *y*^2^_ orbitals of the respective iron. The α and β transitions to the *d*_*x*^2^ – *y*^2^_ are significantly split, which is attributed to spin polarization.^96,107^ In **Q-6**, the same order of orbitals but more overlap of the signals is found. The transitions into the t_2g_ set are again found at lowest energy, followed by the transition into the Fe2 *d*_*z*^2^_ orbital. But here, the transitions into the Fe1 *d*_*z*^2^_ orbital is found between the α and β transition to the *d*_*x*^2^ – *y*^2^_ orbitals. Using the assignment given by the XAS spectra, open- and closed-core model complexes can be compared. In the both models, the excitations with the highest energy are excitations into the unoccupied *d*_*x*^2^ – *y*^2^_ orbitals of the α and β subset. The *d*_*z*^2^_ orbitals come at lower energy, and the t_2g_ orbitals, at the lowest. This has not been observed in open-core model complexes,^27^ although the difference could arise from the difference in spin state. Yet, the spectroscopic trends between model complexes and protein models are very similar, both computationally and in experiment.

#### 3.2.5 Raman Modes of MMOH_Q_

One argument for the proposal of a closed core in MMOH_Q_ has been derived from the experimental resonance Raman spectrum with labeled molecular oxygen.^17^ In the experimental resonance Raman ^16^O–^18^O difference spectra, two signals are observed, one at 690/654 cm^–1^ and the other one at 556/533 cm^–1^. While the latter was attributed to MMOH_T_, which is formed after the reaction of MMOH_Q_ with methane, it should be noted that the 556/533 cm^–1^ signal is also observed, when methane is not present, and the authors suggest these signals may also arrive from the spontaneous decay of Q in the absence of substrate.^17^ We also note that, due the high noise level on the high energy side of the Raman spectrum, additional signals above 800 cm^–1^ may not have been captured. Hence, for the present analysis, we focus on the 690/654 cm^–1^ feature, acknowledging that additional experiments may be needed to positively assess the presence or absence of resonantly enhanced Raman features above 800 cm^–1^.

Further, we acknowledge that the accurate calculation of the resonance Raman spectra of antiferromagnetically coupled transition metal dimers is a challenging undertaking. Given that most of the relevant information is contained in the shifts of the vibrational modes of the chromophore in the core, we have decided to study the nonresonant Raman spectra first in order to identify isotope sensitive vibrations in the various models and their relationship to the features observed in the resonance Raman experiment. As a consequence of this choice, the calculated intensities are of secondary importance for the comparison to experiment. One assumption taken here is that both oxygens from molecular O_2_ are incorporated into the diiron core of MMOH_Q_,^22^ although not necessarily in the μ-oxo bridges.

**Figure 12:**
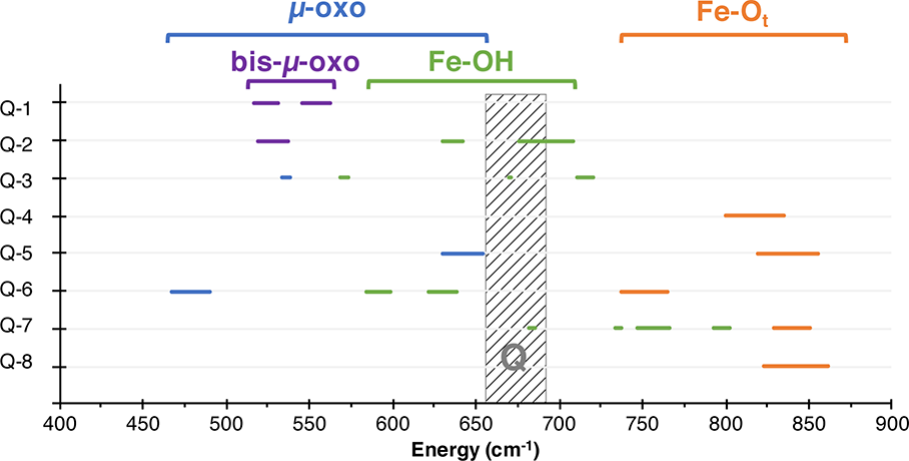
Overview on the calculated Raman frequencies (in cm^–1^). Shifts are indicated as bars to lower frequencies. Modes that are predominantly along the Fe–O_t_ are labeled in orange, modes following Fe–OH are shown in green, Fe−μO–Fe modes are in blue, and Fe–bis-μ-O–Fe modes are in purple. The experimental modes, assigned to MMOH_Q_, are shown as a gray bar.

Based on this reasoning, Raman modes of the constructed models are calculated and their difference spectra after in silico isotope labeling are investigated. However, the frequency shift upon isotope labeling is the same in both the resonant and nonresonant spectra. The resulting spectra are shown in Figure S16. Due to mechanical coupling of the core vibrations with the ligand environment (primarily involving the glutamate carboxyl ligands), the calculated Raman spectra are fairly complex. To partially resolve this issue, partial Hessians involving only the Fe_2_O_2_ core were calculated. There, the “pure” core modes are observed, as shown in Figure S17 which can be identified and then transferred to the full Hessian spectrum. A tabular summary is given in Table S7. The problem of overlapping modes when comparing the ^16^O–^18^O differences has been overcome by using mode tracking analysis. A summary of this analysis is given in Figure 12. Note that an additional layer of complexity in this analysis is that for open-core models of type **Q-6**, several possibilities for the insertion and cleavage of the labeled oxygen exist. Therefore, the three permutations of each of these modes have to be included in the analysis (see Figure S18).

In the following, each class of vibration is analyzed in terms of energy and isotope shifts, summarized in Table 7. Pure Fe–O_t_ stretches are observed around 850 cm^–1^ (model **Q-4**, model **Q-8**), but can be shifted down to 765 cm^–1^ by hydrogen bonding (model **Q-6**, shown in orange in Figure 12). While in model **Q-8** the isotope shift is −39 cm^–1^, in model **Q-7** a shift of −20 cm^–1^ is found. Depending on the combination of labeled oxygen, the isotope shift in model **Q-6** is −4 cm^–1^ or −27 cm^–1^.

**Table 7:** Characteristic Modes in the Non-Resonant Raman ^16-18^O Difference Spectra and Their Respective Shifts, Sorted by the Motif^a^

	^16^O min (cm^–1^)	^16^O max (cm^–1^)
Fe−μO–Fe	490	654
Fe–bis-μ-O–Fe	532	563
Fe–OH	599	802
Fe–O_t_	765	874

a Note that for model **Q-6**, where different labeling patterns were probed, multiple entries to the same mode with different shifts are obtained.

Pure Fe–OH stretches are uncommon, because of the amount of hydrogen bond acceptors around the Fe_2_O_2_ site. The least hydrogen bound example can be found in model **Q-2** at around 700 cm^–1^. This transition has an isotope shift of −33 cm^–1^. Hydrogen bound Fe–OH stretches are observed in model **Q-3** at 720 cm^–1^, with an isotope shift of −10 cm^–1^. Fe−μO–Fe shifts are observed below 500 cm^–1^ (model **Q-6**), but can be found at higher frequencies depending on the involved ligand. Combinations of Fe–OH and Fe−μO–Fe stretches can be found at 654 cm^–1^ in model **Q-6**, with an isotope shift of −24 cm^–1^. This depends on the actual positions of the labels as shown in Figure S18.

Modes involving Fe−μO_2_–Fe are predominantly observed in model **Q-1**, in an energy range between 532 and 562 cm^–1^. These modes can be described as symmetric and asymmetric “breathing” modes. They combine with the glutamate ligand vibrations and hence are quite a bit lower than analogous model complexes. These vibration modes have calculated isotope shifts of −14 and −17 cm^–1^, respectively.

In the range of the experimentally observed MMOH_Q_ signals, between 650 and 700 cm^–1^ two types of signals can be found. These include mostly combination bands between the Fe−μO–Fe stretches (model **Q-5**) and Fe–OH stretches (model **Q-2**). These modes have an isotope shift of −25 and −32 cm^–1^, respectively (see Figure S19 for depiction of these modes). Larger isotope shifts, like the 36 cm^–1^ observed in the experimental MMOH_Q_ signal from 690 to 654 cm^–1^, are only observed for Fe–O_t_ stretches between 650 and 700 cm^–1^, for example for model **Q-5**, model **Q-6**, and model **Q-8**. For the Fe−μO–Fe and Fe–OH combination bands that lie in the experimental MMOH_Q_ range, maximum shifts of −18 to −25 cm^–1^ are observed.

In conclusion, hydrogen bonding and its modulation of the Raman modes are an essential aspect of the MMOH_Q_ Raman spectra. In the presented models, no perfect agreement with the experimental energy range and isotope shift was found. It seems plausible that the experimentally observed features can be attributed to a combination of an Fe–OH and Fe−μO–Fe mode.

#### 3.2.6 Combining Evidence on MMOH_Q_

A summary of all previously discussed criteria for the MMOH_Q_ models is given in Table 8. From the geometry analysis it was shown that only open core models agree with the revisited EXAFS distance,^28^ albeit the μ-oxo bridged models are 0.2 Å too short. Among the open models, models **Q-6** and **Q-8** showed the best agreement in the Mössbauer simulations. In the analysis of XAS pre-edges, it was shown that only models with terminal oxo groups reproduce the intense pre-edge features. Hydrogen bonding modulates the intensity and position of the pre-edge, which suggests that there are many more options than the selection presented here. The best agreement for IWAE, area, and pre-edge shape is observed for models **Q-5** and **Q-6**. In the Raman experiment, the best agreement for isotope shifts is observed for terminal oxo stretches, while signals in the experimental range originate from terminal hydroxo stretches in **Q-2** or μ-oxo stretches in **Q-5**. It was shown that hydrogen bonding allows for the modes spanning the whole diiron core, which improves agreement with experiment in terms of energy and isotope sensitivity. Combining the results from Mössbauer, XAS, and Raman calculations with the analysis of the geometry indicates that model **Q-6** is the structure that is most consistent with the experimental data for MMOH_Q_.

**Table 8:** Summary of the Experimental Criteria for the Evaluation of the MMOH_Q_ Models^a^

	Geometry	Mössbauer	XAS pre-edge		Raman	
model	Δ*d*(Fe–Fe) (Å)	Δδ (mm/s)	ΔIWAE (eV)	Area Ratio	Δν (cm^–1^)	Δδ (cm^–1^)
**Q-1**	–0.60	**0.01**	–0.87	0.36	–127.27	–19.26
**Q-2**	–0.56	0.79	0.57	1.46	18.25	**-3.17**
**Q-3**	–0.42	0.33	–0.10	**0.99**	30.65	–25.63
**Q-4**	–0.37	0.54	**–0.04**	1.57	144.72	**-1.28**
**Q-5**	**-0.20**	0.26	**-0.01**	1.45	–35.74	–11.21
**Q-6**	**-0.19**	**0.10**	–0.79	**1.10**	–51.05	–18.19
**Q-7**	0.45	**0.07**	–0.16	1.49	**–4.16**	–32.41
**Q-8**	0.28	**0.05**	**–0.08**	2.05	171.85	**2.78**

a Values in bold indicate agreement with experiment within the target accuracy as established before.

### 3.3 Discussion

In the following we discuss the extent to which the MMOH_Q_ models developed in this work can be compared with synthetic high valent model complexes,^27,34,105^ and we explore the possible implications of the present results for the reaction mechanism of sMMO.

#### 3.3.1 Comparison to Model Complexes

Biomimetic model complexes can be used as structural models, to explore the electronic structure of the diiron core, or as functional models, stabilizing relevant Fe(IV) oxo motifs to investigate the reactivity and plausible mechanisms. Structural models^105^ (Figure S1) have been discussed extensively in connection with the XAS pre-edge analysis.^27^ It should be recalled however that the spin states of the irons in these model complexes do not necessarily reflect the electronic structure of the protein, as they are all *S* = 1. Hence, only some of the findings in the synthetic complexes are transferable to our comparisons between closed- and open-conformations of the QM/MM models. One common feature is the orientation of the *d*_*z*^2^_ orbital in the closed-core QM/MM models (**Q-1**) and closed-core model complexes (complex 3 in ref (27)). Resulting from this agreement in the electronic structure, also a reasonable agreement in the spectroscopic properties is observed:^27^ both **Q-1** and the closed-core model complex feature a low intensity pre-edge, with comparable IWAE. The Mössbauer results indicate two similar irons in both cases.

In the case of the open-core QM/MM models and the open-core model complex (Figure S1, model 4),^27^ a direct comparison is more complicated. One similarity is the behavior of the *d*_*z*^2^_ orbitals of the two irons in QM/MM models and model complexes. In both cases the *d*_*z*^2^_ orbital of the individual irons are orthogonal to each other. However, since the terminal oxo ligand in **Q-5** is oriented in the same way as the terminal water ligand in **Q-1**, no changes in the orientation of the *d*_*z*^2^_ orbital were found here. Instead, the *d*_*z*^2^_ orbital of the other iron, Fe2, is oriented along the Fe-μ-oxo/Fe-OGlu209 bond. As discussed for the model complexes, this orthogonality behavior is controlled by the strongest σ type metal–ligand interaction. In the model complexes, this is unambiguously the Fe=O σ interaction. However, in the protein the glutamate oxygens compete with the μ-oxo and terminal oxo ligands. Hence, this reordering is not observed for all open QM/MM models. Another difference to the synthetic diiron Fe(IV) complexes is the *d* orbital order for the iron sites. In the synthetic model the identity of the LUMO differs for the two irons, whereas in the QM/MM open-core models both sites share the same identity of the LUMO. One possible explanation could be the similarity of the two iron sites in the synthetic models, as reflected by Mössbauer spectroscopy: here the open-core model complex shows two very different irons, while the QM/MM models of the enzymatic active site (**Q-5** and **Q-6**) show very similar irons. In conclusion, the open-core QM/MM models of MMOH_Q_ and the available synthetic open-core analogues are less comparable than the closed-core versions and hence the transferability of observations based on synthetic open-core complexes to the actual enzyme should be considered with caution.

Aside from these structural models, the reactivity of high valent iron complexes has been studied in great detail on mononuclear iron(IV) complexes. Of course, with two irons, MMOH differs from the models discussed in these studies. However, for the open-core models of MMOH_Q_ the *d*_*z*^2^_ orbital is oriented along the Fe–O moiety, making it comparable to the mononuclear iron complexes. Yet the fact that the *d*_*z*^2^_ is orthogonal to the other moiety may have implications in the reactivity or selectivity. The CH reactivity was probed using model complexes featuring different iron oxo units. A steep increase in reactivity for the models with an Fe(IV)=O moiety was observed.^34^ While Fe(IV) diiron complexes are rare,^34,110^ mononuclear Fe(IV) intermediates are studied extensively,^111^ especially concerning the influence of the iron spin states on the reactivity.^112,113^ One central question is the influence of the electronic structure at the Fe–O moiety on the reactivity and mechanism of the C–H abstraction. For one, depending on the local spin state of the iron, the substrate interacts with either a triplet or quintet ground state. On the other hand, the local geometry of the electron accepting orbitals at the iron leads to different possibilities as to how the reaction can be rationalized.^114^ Depending on whether these are of σ- or π-type, these are referred to as σ- or π-channels.^115^ If the electron is transferred into the iron *d*_*z*^2^_, which can be understood as σ-antibonding in the Fe–O bond, this is referred to as the σ-channel. In the quintet state, the *d*_*z*^2^_ orbital is the HOMO; hence it is energetically favorable to transfer the electron here. If on the other hand, the electron is inserted into the iron *d*_*xz*_ or *d*_*yz*_ orbitals, this is referred to as the π-channel. These are the SOMOs in the triplet state and, hence, preferred targets here. Both channels lead to different transition state geometries, i.e. a linear or bent Fe–O-substrate coordinate. Such detailed studies are beyond the scope of the present study. However, the established concepts can be placed in the context of our present study and combined with existing discussion on the MMOH mechanism.

#### 3.3.2 Implications for Reaction Mechanisms Involving MMOH_Q_

In the following, the mechanism of the MMOH active site is discussed, as far as MMOH_Q_ is involved. A detailed computational investigation of possible mechanisms is outside the scope of the present work and will be reported in future studies. While the results presented above are in favor of an open-core assignment for MMOH_Q_, the majority of mechanistic ideas and computational mechanistic studies in the literature have been discussed on the basis of a closed-core configuration for MMOH_Q_. Hence, these are reviewed before we discuss the implications of an open core for MMOH_Q_.

The structure of the MMOH_Q_ has immediate implications for the catalytic steps leading to the formation of MMOH_Q_ and for methane activation. MMOH_Q_ is formed by decay of the MMOH_P_, the exact structure of which remains a subject of controversy. MMOH_P_ is formed by O_2_ insertion of MMOH_red_. There are several plausible motifs for MMOH_P_,^8^ as shown in Scheme S1. Similar to the structure, the mechanism of MMOH_P_ to MMOH_Q_ conversion is under debate.^8,24^ Yet, based on the information gained on MMOH_Q_, specific possibilities have been formulated on how the transition from MMOH_P_ to MMOH_Q_ might occur (Scheme S1). Based on analogy to the cytochrome P450 monooxygenase,^116^ a similar mechanism (**I**) was proposed for MMOH.^117^ However, this is in conflict with the resonance Raman experiment^22^ showing that both oxygens from molecular oxygen are incorporated into the diiron core of MMOH_Q_. Yet, it was found that the proton transfer was not part of the rate-determining step,^118^ which led to the proposal of a proton independent mechanism (**II**). Upon reexamining the role of protons in the MMOH_P_ to MMOH_Q_ transition, it was found that proton transfer promotes O–O bond cleavage,^119^ upon which a matching mechanism was proposed (**III**). These have been modeled computationally.^52,68^ Comparisons to other nonheme iron enzymes were made.^39^

All of these considerations assume a bis-μ-oxo core for MMOH_Q_. However, it is plausible that these mechanisms could result in an open-core geometry, depending on how the O–O bond is broken and how the oxygens reassemble. Homolytic cleavage of the oxygen bond in a trans bound O_2_ leads to a bis-μ-oxo core, while heterolytic cleavage of the O–O bond might result in a terminal-oxo/μ-oxo core.

Similar considerations can be made for the methane activation step or MMOH_Q_ decay. It is established by kinetic studies that other steps than the C–H cleavage are rate limiting in the enzymatic reaction.^22^ The large kinetic isotope effect observed for the methane activation step was interpreted as tunneling of the hydrogen atom during the abstraction process.^120,121^ So far there is evidence for two types of mechanisms: radical mechanisms^50,122−128^ or concerted mechanisms.^129−131^ These were further analyzed using computational studies. The results can be summarized into iron-centered and oxygen-centered mechanisms (Scheme S2). Mechanism **IVa** is characterized by rearrangement of the bis-μ-oxo core into an Fe(IV) oxo radical, which then abstracts a proton from the substrate. The remaining CH_3_ radical is then coordinated to the iron, and eventually recombined to methanol. This mechanism was suggested by Siegbahn and co-workers.^35,36,38^ One could also imagine an oxygen centered variance of this mechanism, then termed the radical rebound mechanism: **IVb**.^36,38,51^ Here, a CH bond of the substrate is broken by a μ-oxo, leading to a CH_3_ radical that rebounds with the same oxygen. Mechanism **Va**, advocated by the Yoshizawa group,^42−49,69^ is best described as a concerted nonradical mechanism, initiated by a proton transfer to a μ-oxo while stabilizing the CH_3_ group at the iron, before recombination to methanol. A variant of that mechanism was termed the nonsynchronous concerted pathway (**Vb**), explored by Friesner and Lippard.^61,64,65,131^ Here, the CH_3_ group is stabilized by the protein environment, not the iron. Hence this mechanism can be viewed as oxygen-centered.

For the open-core models in this study, several of these mechanisms are plausible. The spontaneous opening of the μ-oxo bond is less likely for models with only one μ-oxo bond, especially for the glutamate bridging ligands. The other μ-oxo centered mechanisms could be transferred to the mono-μ-oxo ligands as well. However, as the open models feature terminal oxo ligands, it is highly likely that the terminal oxo is a central aspect of the mechanism, as it is in model complexes.

**Scheme 1:**
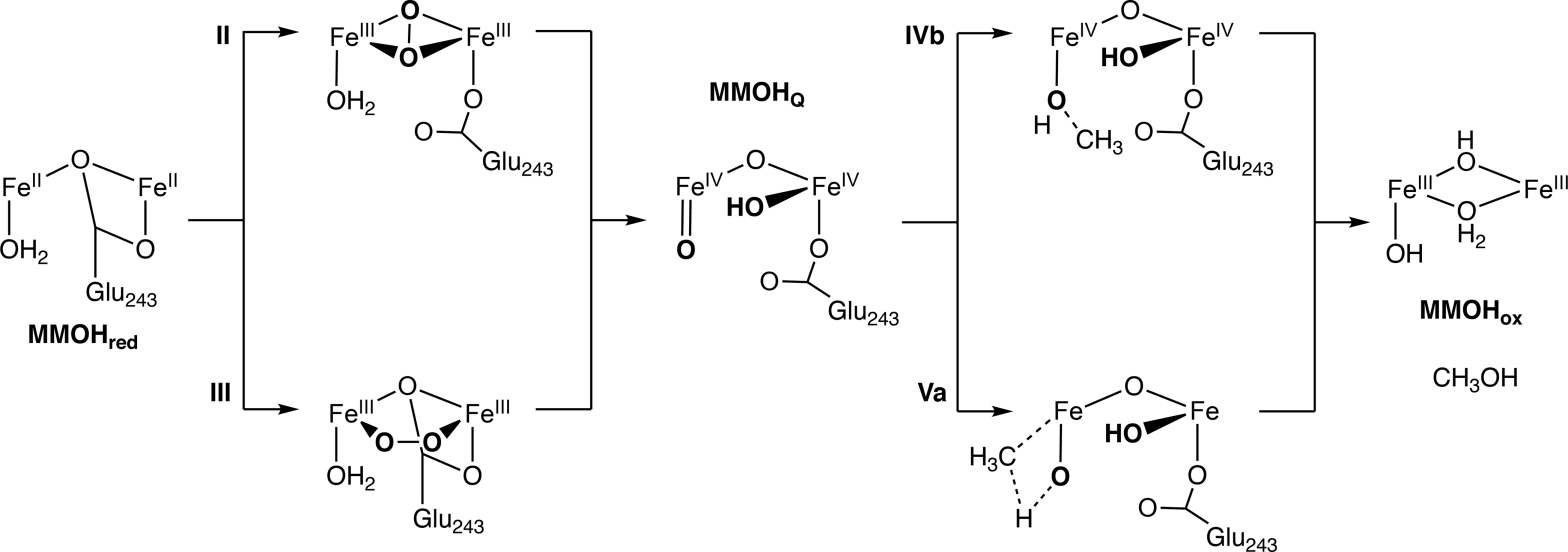
Possible MMOH_red_ to MMOH_ox_ Transitions for Open Cores of MMOH_Q_ Note that for transition states explicit steps are omitted for clarity.

For the presented case of an open model, possible mechanisms are collected in Scheme 1. Upon heterolytic cleavage of the dioxygen bond, the terminal oxo ligand is formed. The bridge could be protonated either during the cleavage or subsequently. Similar to model complexes the terminal oxo is expected to be the reactive group in the methane activation step, following a similar reactivity as for the mononuclear iron complexes. Both a radical mechanism, based on the oxyl character of the Fe=O group, or a concerted mechanism seem plausible for the open models. In computational studies of model complexes, it was found that the reactivity of the Fe(IV)=O unit is modulated by the ligand to the second iron,^132^ similar to what has been discussed for the Raman calculations before. Further examination of these distinct possibilities will be pursued in the future using the here developed QM/MM setup.

## 4 Conclusions

This study presented a wide range of possible models for the MMOH_ox_ and MMOH_Q_ intermediates of soluble methane monooxygenase, focusing on elucidating in great detail the possible connections between structural features and spectroscopic properties. The approach followed in this work emphasizes the construction of high-level QM/MM models that explicitly take into account the protein environment, combined with high-level quantum chemical calculations of spectroscopic parameters that enable reliable evaluation of the computational models against experimental information on the distinct catalytic intermediates. We have evaluated models for both intermediates on the basis of experimental geometric constraints, Mössbauer spectroscopy, and X-ray absorption spectroscopy. Specifically for MMOH_Q_ we have also studied in detail the vibrational properties of the different computational models in connection to resonance Raman data.

A central question concerning the nature of MMOH_Q_ is the structural type of the diiron core, i.e. whether it is a closed core, with at least two bridging oxo groups leading to an Fe_2_O_2_ “diamond” topology, or whether it adopts a more open-core configuration, which has been associated in synthetic model chemistry with higher reactivity. A major experimental constraint is the Fe–Fe distance of MMOH_Q_ as deduced by recent EXAFS measurements, which revised the incorrect short distance assumed in prior studies. We demonstrate that the EXAFS distance agrees only with open-core QM/MM models of MMOH_Q_. The simulation of the Mössbauer parameters shows agreement for several open-core models. Here, the local geometry of the iron and the hydrogen bonding between the bridges and surrounding amino acids are shown to have a great effect. Analysis of XAS pre-edge data confirms that a closed core without terminal oxo ligands does not lead to the observed pre-edge intensity. With terminal oxo ligands, the necessary intensity is achieved. Here again, the protonation state is of great importance to the shape and intensity of the pre-edge. Importantly, we show that the previous analysis of the resonance Raman experiment was limited in its initial assumptions,^22^ and that the data can be fully consistent with an open-core formulation of MMOH_Q_ if hydrogen bonding between the terminal Fe ligands is considered.

Overall, the present analysis points squarely toward an open-core topology for the MMOH_Q_ intermediate. It offers concrete reinterpretations of experimental data, reconciling suggestions that until now seemed to be in conflict. Moreover, the spectroscopically consistent open-core structure of MMOH_Q_ is finally brought in line with the enhanced reactivity of this type of core, as observed in synthetic analogues. The present conclusions and computational models will form the basis for more reliable investigations of the catalytic mechanism of the enzyme in the future.

## Supporting Information Available

Figures S1–S19, Tables S1–S7, Schemes S1 and S2 (PDF)
