# Supplementary material for: Structure–Spectroscopy Correlations for Intermediate Q of Soluble Methane Monooxygenase: Insights from QM/MM Calculations
Source: J Am Chem Soc. 2021 Apr 22;143(17):6560–77. doi: 10.1021/jacs.1c01180 (PMC8154522; doi:10.1021/jacs.1c01180)
Supplement: Supplementary file 1 — ja1c01180_si_001.pdf [file ja1c01180_si_001.pdf]

SUPPORTING INFORMATION

Structure–Spectroscopy Correlations for Intermediate Q of Soluble Methane Monooxygenase: Insights from QM/MM calculations

Christine E. Schulz,[a] Rebeca G. Castillo,[b] Dimitrios A. Pantazis,[a] Serena DeBeer,[b] Frank Neese [a]

[a] Max-Planck-Institut für Kohlenforschung, Kaiser-Wilhelm-Platz 1, 45470 Mülheim an der Ruhr, Germany.

[b] Max Planck Institute for Chemical Energy Conversion, Stiftstr. 34-36, 45470 Mülheim an der Ruhr, Germany.

Table S1. Available crystal structures of MMOH and Fe-Fe distance for $MMOH_{ox}$ species.

XRD	resolution	year	organism	Fe_1-Fe_2	comment
1FYZ	2.15	2001	Mc		reduced
1FZ0	2.07	2001	Mc	3.40	mixed valence
				3.38	
1FZ1	1.96	2001	Mc	3.18	pH 7, O_a formate
				3.16	pH 7
1FZ2	2.15	2001	Mc	3.41	O_a missing
				3.42	O_b missing
1FZ3	2.03	2001	Mc	3.15	pH 6.2, O_a formate
				3.16	pH 6.2
1FZ4	2.38	2001	Mc	3.11	pH 8.5, Oa formate
				3.00	pH 8.5, Oa missing
1FZ5	2.40	2001	Mc		only one Fe per MMOH
1FZ6	2.05	2001	Mc	2.99	soaked in CH_3OH
				3.04	soaked in CH_3OH , O_a methanol
1FZ7	1.96	2001	Mc	3.09	soaked in C_2H_6, O_a formate, Ob missing
				3.16	soaked in C_2H_6, O_a formate
1FZ8	2.10	2001	Mc	3.08	soaked in Br_2CH_2, O_a missing
				3.01	soaked in Br_2CH_2, O_a missing
1FZ9	2.30	2001	Mc	2.85	cocrystallized with C_2H_5I, O_a, O_b missing
				2.94	cocrystallized with C_2H_5I, O_a missing
1FZH	2.60	2001	Mc	3.10	pressurized with Xe gas, O_a missing
				3.08	pressurized with Xe gas, O_a missing
1MHY	2.00	1996	Mt	2.99	
1MHZ	2.70	1996	Mt	3.07	
1MMO	2.20	1994	Mc	3.46	O_a acetate

				3.36	
1MTY	1.70	1997	Mc	3.04	
				3.14	
1XMF	2.31	2004	Mc		Mn(II) soaked apo-MMOH
1XMG	2.10	2004	Mc		apo-MMOH
1XMH	2.32	2004	Mc		Co(II) reconstituted MMOH
1XU3	2.30	2005	Mc	2.85	soaked with BrC_6H_5, O_a, O_b missing
				2.81	soaked with BrC_6H_5, O_a, O_b missing
1XU5	1.96	2005	Mc	3.07	soaked with C_6H_6
				3.04	soaked with C_6H_6
1XVB	1.80	2005	Mc		soaked with 6-Br$(CH_2)_6$OH
1XVC	2.00	2005	Mc	3.04	soaked with 8-Br$(CH_2)_8$OH
				3.02	soaked with 8-Br$(CH_2)_8$OH
1XVD	2.30	2005	Mc	2.77	soaked with 4-FC_6H_5, O_a, O_b missing
				2.57	soaked with 4-FC_6H_5, O_a, O_b missing
1XVE	2.40	2005	Mc		alcohol bridging Fe
1XVF	2.00	2005	Mc		alcohol bridging Fe
1XVG	1.96	2005	Mc		alcohol bridging Fe
4GAM	2.90	2012	Mc		MMOH + MMOB

Mt: *Methylosinus trichosporium*, Mc: *Methylococcus capsulatus* str. Bath

Analysis of XRD structures.

A careful selection is necessary because in some of the XRD models only one iron is resolved, or bridging oxygens are missing entirely. In others, the bridging ligands are alcohol derived, although in wild type $MMOH_{ox}$ structures it is established, that the bridging ligands are water derived. Therefore, the available structures were sorted according to the following criteria:

(I) The active site contains two iron atoms.

(II) The irons are bridged by two oxygen ligands.

(III) The two oxygen ligands are derived from water, i.e not alcohol, formate or other extrinsic molecules. This also excludes purely glutamate bridged structures, as found in $MMOH_{red}$.[1]

These criteria are purely structural. On the other hand, the crystal structures come with meta information, such as the resolution of the structure or the assigned oxidation state, which was not used as a primary criterium to exclude structures from the analysis. From the original 28 structures collected in Table S1, only 9 fulfill these criteria, which are presented in Table S2. Among them is the 1MHZ[2] structure, which has a resolution of 2.7 Å, as well as the 1FZ0[1] structure, which has been labeled as a Fe(II)Fe(III) mixed valence state. All other structures are labeled as Fe(III)Fe(III), and therefore can be compared to $MMOH_{ox}$.

The primary target of this comparison is the diiron core geometry, particularly the distance between the irons. In Figure 3 of the main text it is evident that the distance count shows a clustering of distances between 2.99 and 3.16 Å, with two outliers at 3.38 and 3.40 Å from the mixed valence 1FZ0[1] structure. This confirms that the crystal structure average of the diiron distances is oxidation state specific. If the mean distance is calculated without the outliers, it shortens to 3.07 ± 0.06 Å. This is in excellent agreement with the HERFD-EXAFS distances of 3.06 Å[3] and previous EXAFS.[4] The second aspect of the Fe_2O_2 core is the distance between iron and the bridging oxygen ligands. The Fe-O_a distances show a narrow distribution, while the Fe-O_b distances are distributed broadly. The mean Fe-O_a distance is 1.85 ± 0.13 Å.

The mean Fe-O_b distance is 2.48 ± 0.29 Å. Again, two outliers can be found, at 3.07 and 3.22 Å. The outliers again belong to the 1FZ0 structure, and originate from the same Fe_2O_2 site. Without these two, the mean Fe-O_b distance is 2.41 ± 0.22 Å.

Table S2. Overview of crystal structures with intact Fe_2O_2 cores. Labeling according to Figure 2. Resolutions and distances are in Å.

PDB ID	Res.	year	Fe-Fe	Fe-O_t	Fe_1-O_a	Fe_2-O_a	Fe_1-O_b	Fe_2-O_b
1MHY[2]	2.00	1996	2.99	2.23	2.04	1.71	2.17	2.15
1MHZ[2]	2.70	1996	3.07	2.41	1.79	2.18	1.93	2.45
1MTY[5]	1.70	1997	3.04	2.28	1.78	1.94	2.43	2.58
			3.14	2.27	1.62	2.02	2.24	2.51
1FZ0[1]	2.07	2001	3.40	2.50	1.60	1.82	2.54	3.07
			3.38	2.40	1.70	1.79	2.58	3.22
1FZ1[1]	1.96	2001	3.16	2.44	1.81	1.95	2.71	2.79
1FZ3[1]	2.03	2001	3.16	2.41	1.99	1.98	2.39	2.78
1FZ6[6]	2.05	2001	2.99	2.35	1.86	1.85	2.18	2.18
1XU5[7]	1.96	2005	3.07	2.19	1.82	1.80	2.16	2.48
			3.04	2.14	1.82	1.80	2.31	2.54
1XVC[7]	2.00	2005	3.04	2.11	1.87	1.98	2.43	2.60
			3.02	2.36	1.72	1.85	2.49	2.56
Average			3.12	2.31	1.80	1.90	2.35	2.61
w/o 1FZ0			3.07	2.29	1.83	1.91	2.31	2.51

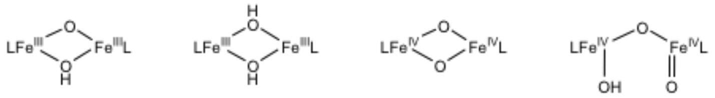

Model	1	2	3	4
S_{tot}	0	0	0	2
S_{loc}	5/2,5/2	5/2,5/2	1,1	1,1

L:

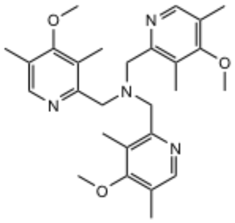

Figure S1. Overview on the molecular model complexes used for the XAS extrapolation and comparison.

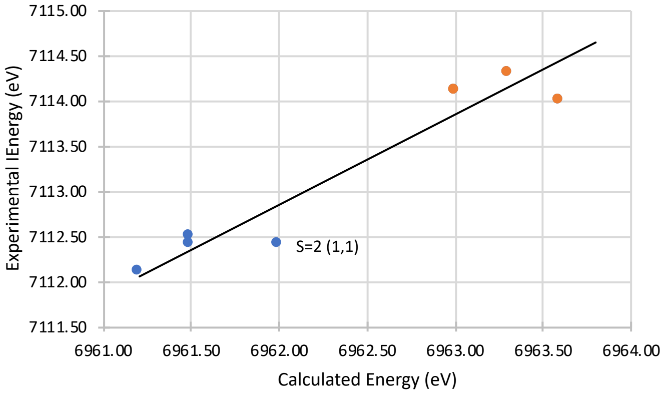

model	ΔEnergy (eV) p1	p2
1	150.90	150.40
2	151.00	151.00
3	150.90	151.10
4	150.40	151.10
AVG	**150.85**	
STD	**0.29**	

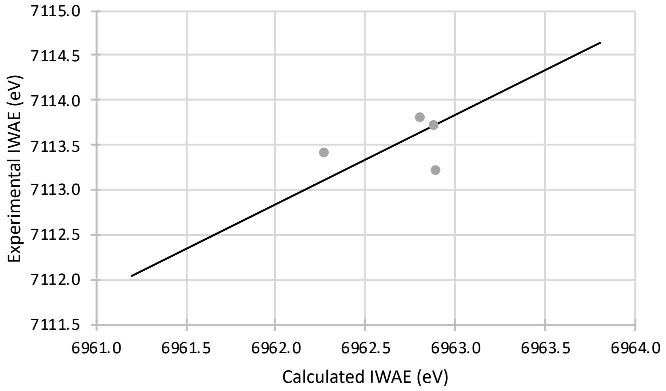

model	ΔEnergy (eV) IWAE
1	150.30
2	151.12
3	150.99
4	150.81
AVG	**150.81**
STD	**0.34**

Figure S2. Linear relationship between the calculated and experimental energy, for the individual peak energies (P1 in blue, P2 in orange, top) and the IWAE (grey, bottom). The numbers for the model complexs correspond to those in reference [8], where the experimental HERFD XAS data were first reported.

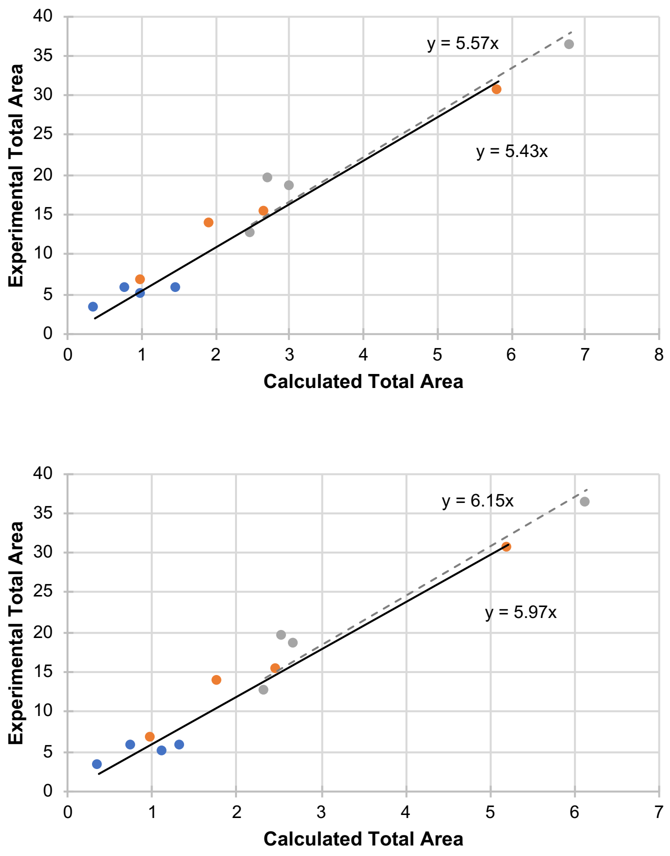

Figure S3. Linear relationship between the calculated and experimental total area, for the individual peak areas (P1 in blue, P2 in orange) and the total area (grey). The regression lines for the sum of the individual peak areas are shown in black, the regression line of the total are is shown in gray dashes. Both regression lines are forced through (0,0). The top panel shows calculated areas determined from refitting the simulated spectra, the bottom panel shows calculated areas determined by calculating the sum of the oscillator strengths.

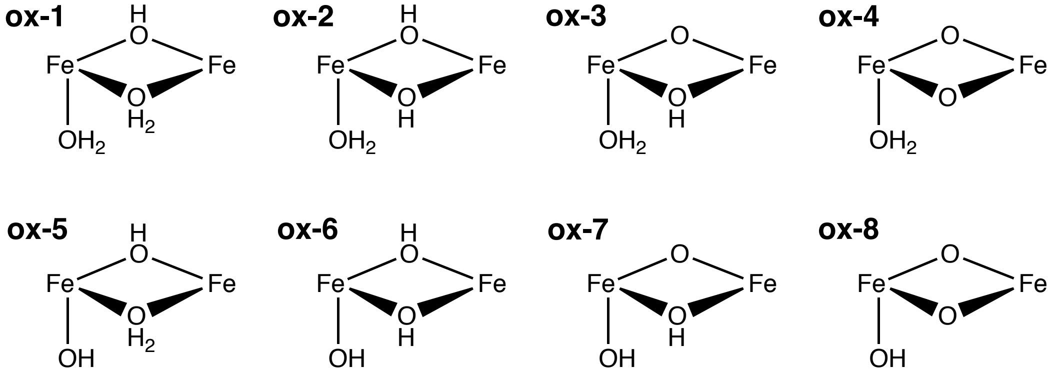

Figure S4. Schematic representation of the possible $MMOH_{ox}$ structures.

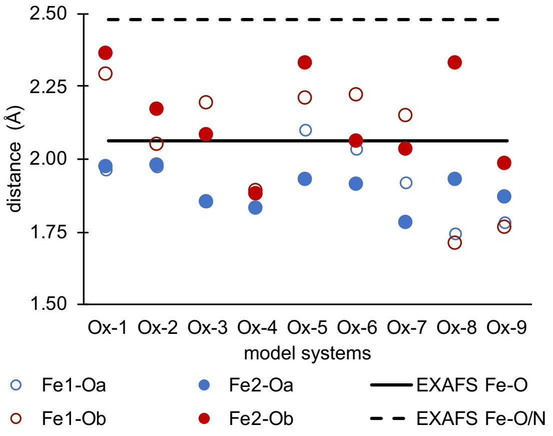

Figure S5. Difference of the Fe-O bond lengths compared to the EXAFS Fe-O and Fe-O/N distances.

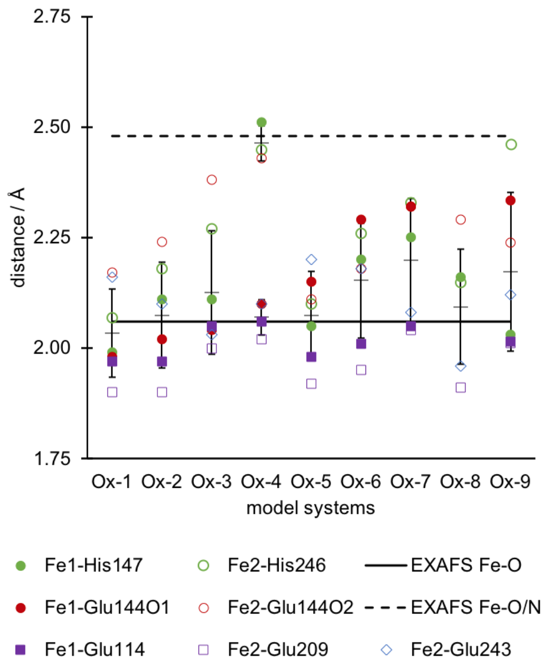

Figure S6. Difference of the Fe-L bond lengths compared to the EXAFS Fe-O and Fe-O/N distances for the $MMOH_{ox}$ models. Averages and standard deviations of the calculated Fe-L distances are given in black bars.

Table S3. Mulliken spin populations and exchange coupling constants for the QM/MM $MMOH_{ox}$ models. The calculated $\hat{S}^2$ expectation values are presented in Table S4.

	Fe1	Fe2	O_a	O_b	O_t	J
**ox-1**	4.23	-4.22	0.01	-0.01	-0.05	-20.8
**ox-2**	4.23	-4.27	0.02	0.00	-0.06	-6.3
**ox-3**	4.17	-4.19	0.02	0.01	-0.05	-30.9
**ox-4**	4.10	-4.10	0.04	-0.02	-0.03	8.0
**ox-5**	4.23	-4.22	0.10	0.00	-0.24	-16.0
**ox-6**	4.24	-4.18	0.11	0.07	-0.38	-7.1
**ox-7**	4.19	-4.26	0.25	0.04	-0.25	-29.3
**ox-8**	3.34	-3.31	0.14	0.37	-0.17	-31.9

Table S4. Calculated expectation values of $\hat{S}^2$ for the high-spin (HS) and broken-symmetry (BS) case for $MMOH_{ox}$.

Model	$< \hat{S}^2_{\mathrm{HS}} >$	$< \hat{S}^2_{\mathrm{BS}} >$
ox-1	30.01	4.97
ox-2	30.01	4.97
ox-3	30.02	4.87
ox-4	30.03	4.86
ox-5	30.01	4.98
ox-6	30.02	3.62
ox-7	30.02	4.89
ox-8	30.10	5.11

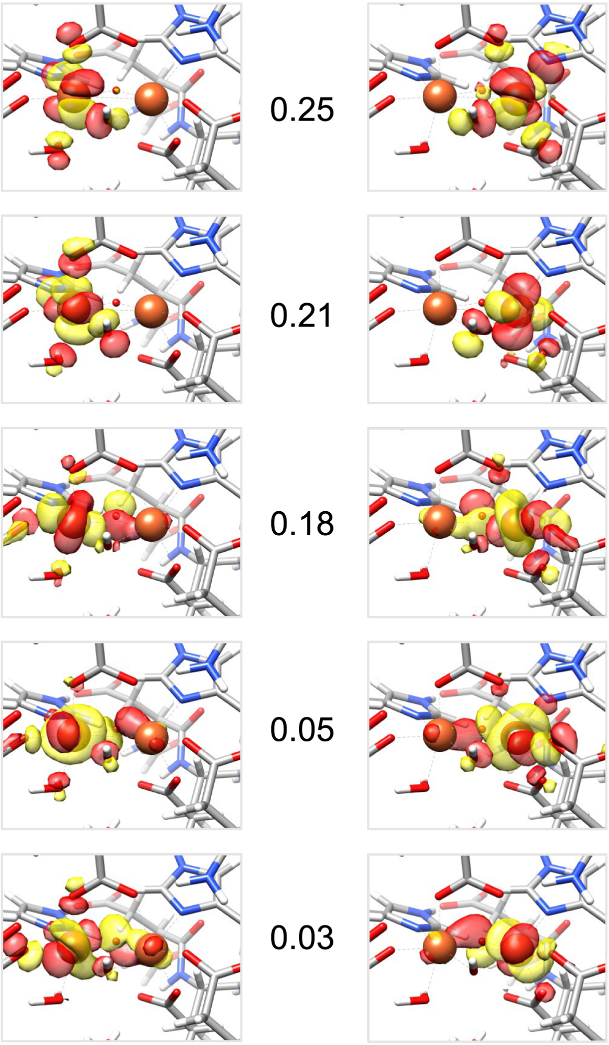

Figure S7. Corresponding orbitals of model **ox-3** and their overlap integrals.

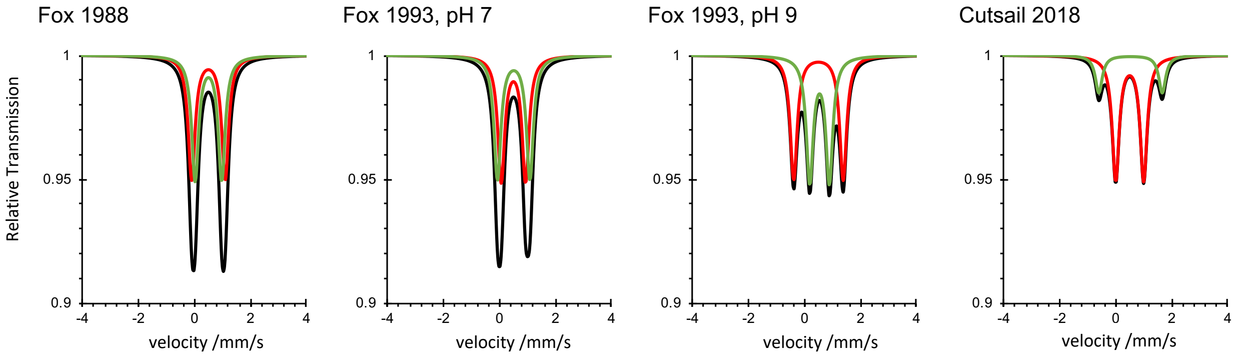

Figure S8. Simulated Mössbauer spectra from experimental parameters given in table 4. Note that for the latest experiment, a signal ratio of 30:70 is used.

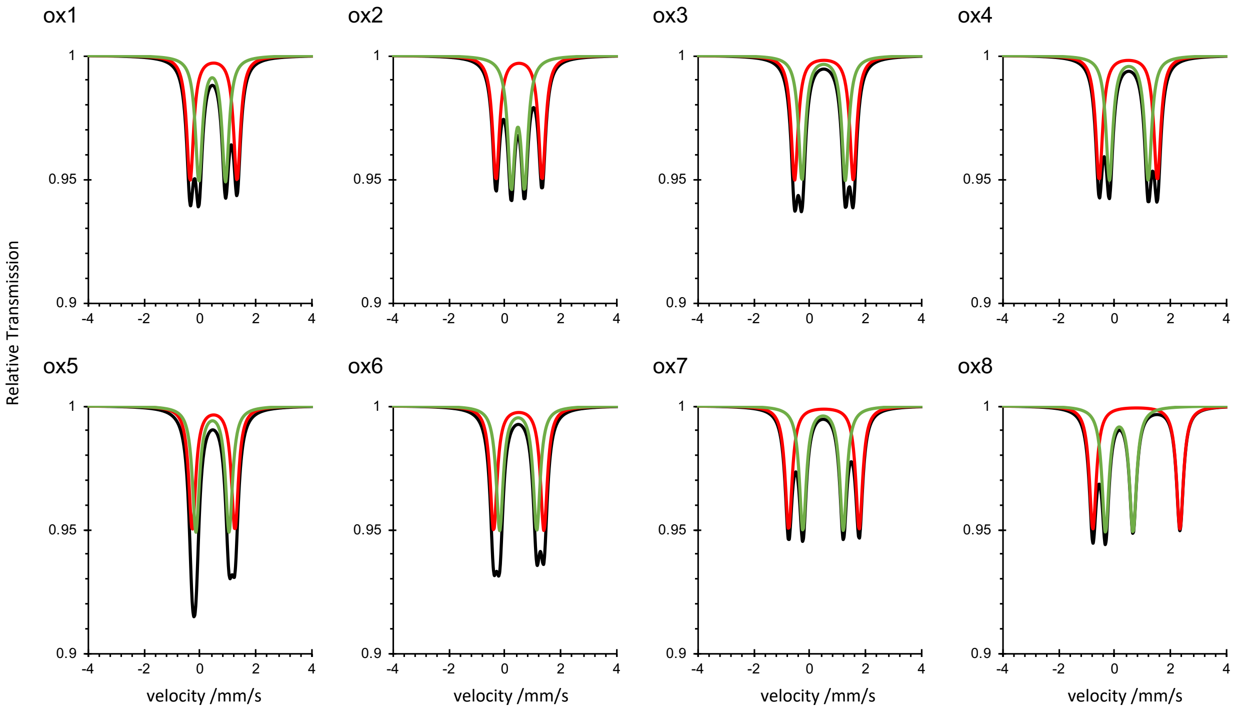

Figure S9. Simulated Mössbauer spectra from the calculated Mössbauer parameters for the MMOHox models, given in table 4.

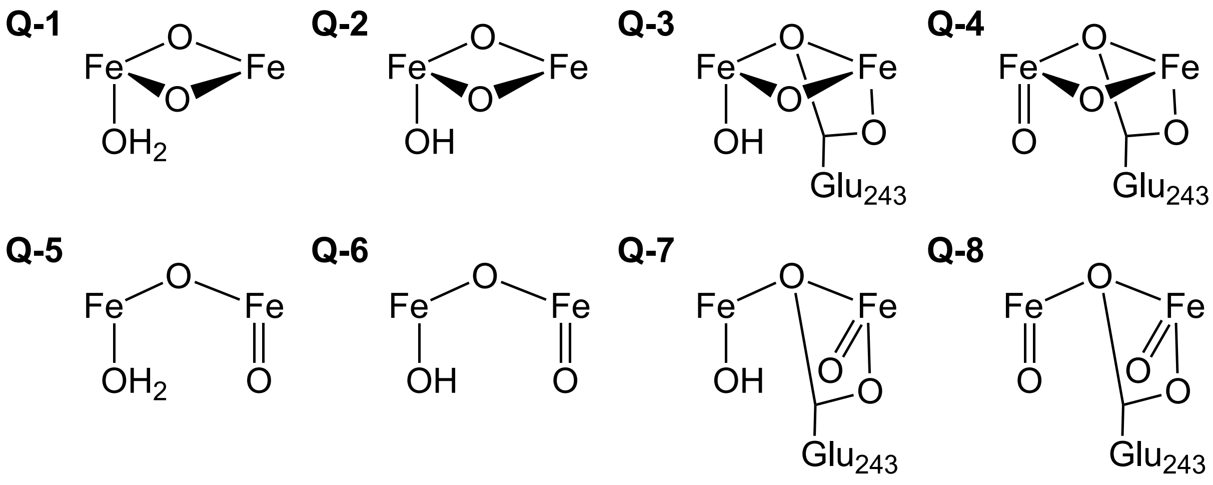

Figure S10. Schematic representation of the possible MMOH$_Q$ structures.

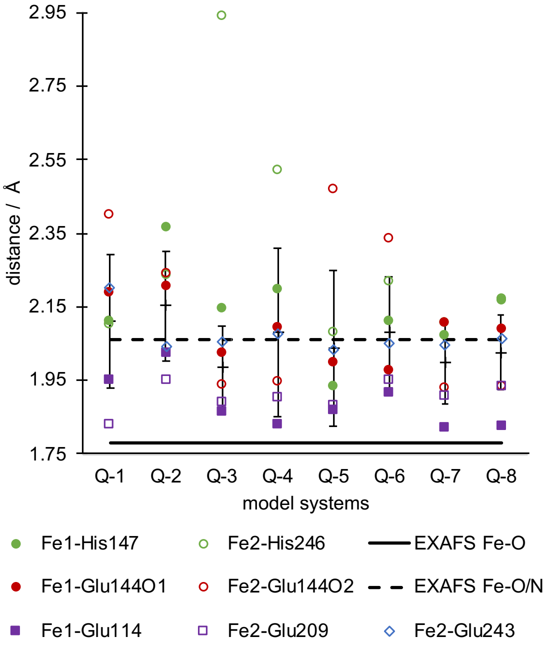

Figure S11. Difference of the Fe-L bond lengths compared to the EXAFS Fe-O and Fe-O/N distances for the MMOH$_Q$ models. Averages and standard deviations of the calculated Fe-L distances are given in black bars.

Table S5. Mulliken spin populations for the QM/MM MMOH$_Q$ models. Note that for open core models (**Q-5** – **Q-8**) instead of O$_b$ the terminal oxygen to Fe$_2$ is given (Fe$_2$-O$_t$). For **Q-3**, **Q-4**, **Q-7**, **Q-8** : O$_a$ = Oε-Glu243, for **Q=5-Q-8**: O$_b$ = O$_t$ at Fe$_2$. The calculated $\hat{S}^2$ expectation values are presented in Table S6.

	Fe$_1$	Fe$_2$	Fe$_1$-O$_t$	O$_a$	O$_b$
**Q-1**	-3.67	3.60	-0.07	-0.03	-0.02
**Q-2**	-2.52	3.12	0.00	-0.36	-0.37
**Q-3**	-3.46	4.16	-0.03	0.00	0.11
**Q-4**	-1.97	2.97	-0.88	0.04	-0.21
**Q-5**	3.10	-3.58	0.60	-0.18	0.00
**Q-6**	-3.23	3.39	-0.49	0.25	0.06
**Q-7**	-3.58	3.24	-0.29	-0.02	0.54
**Q-8**	-3.15	3.24	-0.63	0.02	0.51

Table S6. Calculated expectation values of $\hat{S}^2$ for the high-spin (HS) and broken-symmetry (BS) case for $MMOH_Q$.

Model	$< \hat{S}^2_{\mathrm{HS}} >$	$< \hat{S}^2_{\mathrm{BS}} >$
Q1	20.27	2.50
Q2	20.11	3.21
Q3	20.93	4.96
Q4	20.08	3.26
Q5	20.19	4.15
Q6	20.12	2.94
Q7	20.25	4.25
Q8	20.13	4.13

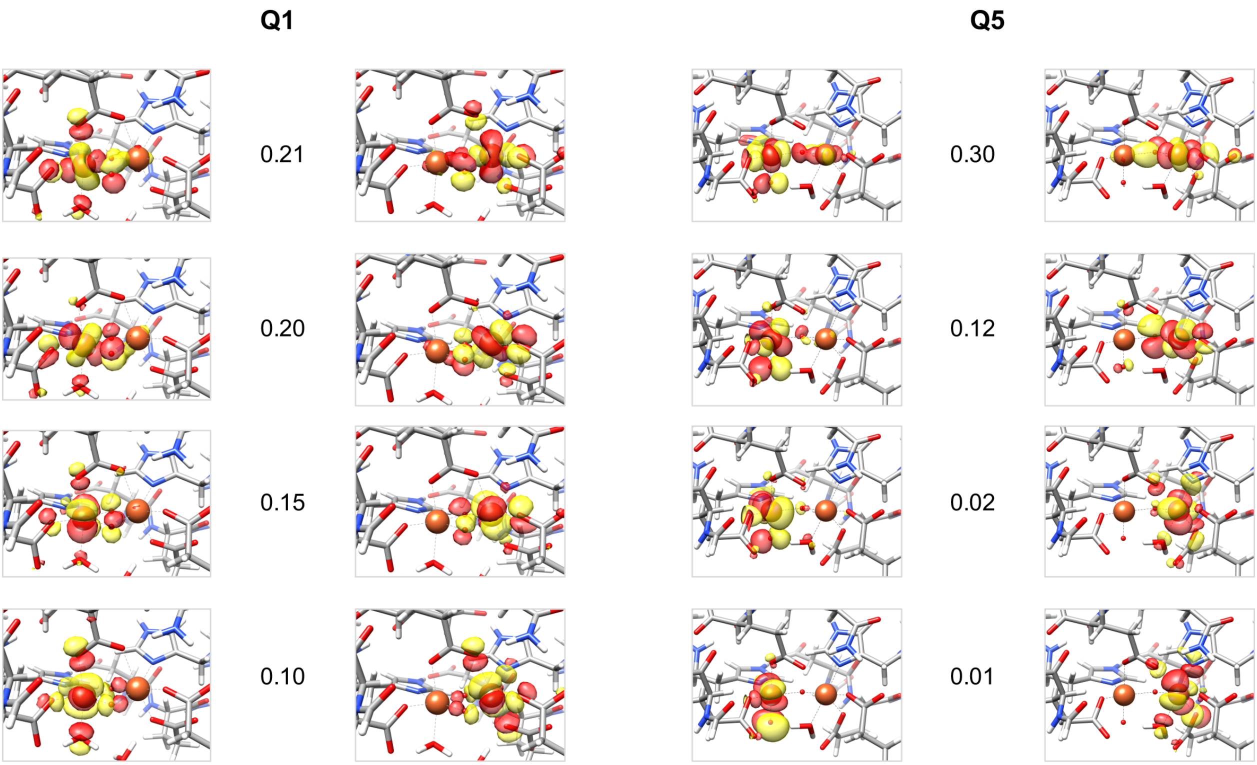

Figure S12. Corresponding orbitals of models **Q-1** and **Q-5** and their overlap integrals.

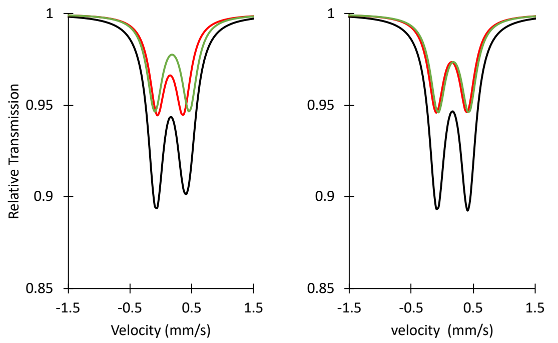

Figure S13. Simulation of the experimental Mössbauer parameter (a) with experimental quadrupole splitting and (b) with an average splitting of 0.5 mm/s.

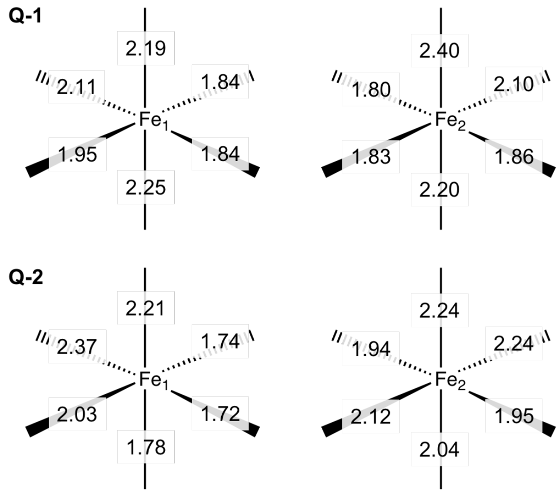

Figure S14. Direct comparison of $MMOH_Q$ models **Q-1** and **Q-2**.

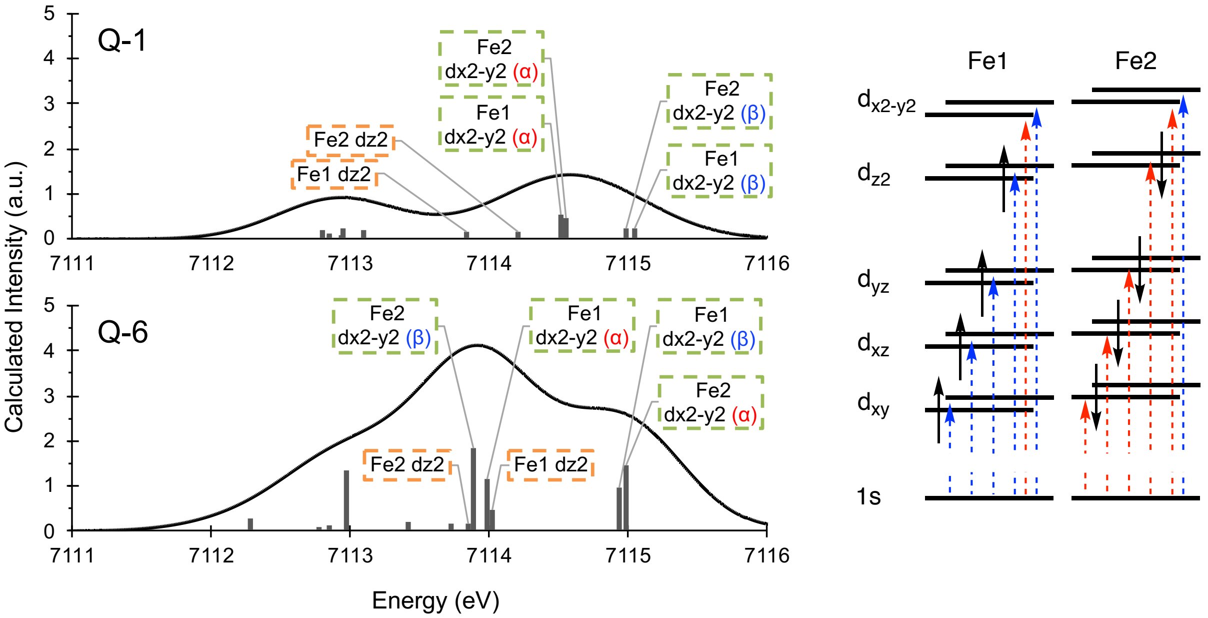

Figure S15. Assignment of the calculated pre-edge XAS spectrum of model **Q-1** vs **Q-6** (left) and schematic representation of the involved transitions (right), with α transitions shown in red and β transitions shown in blue.

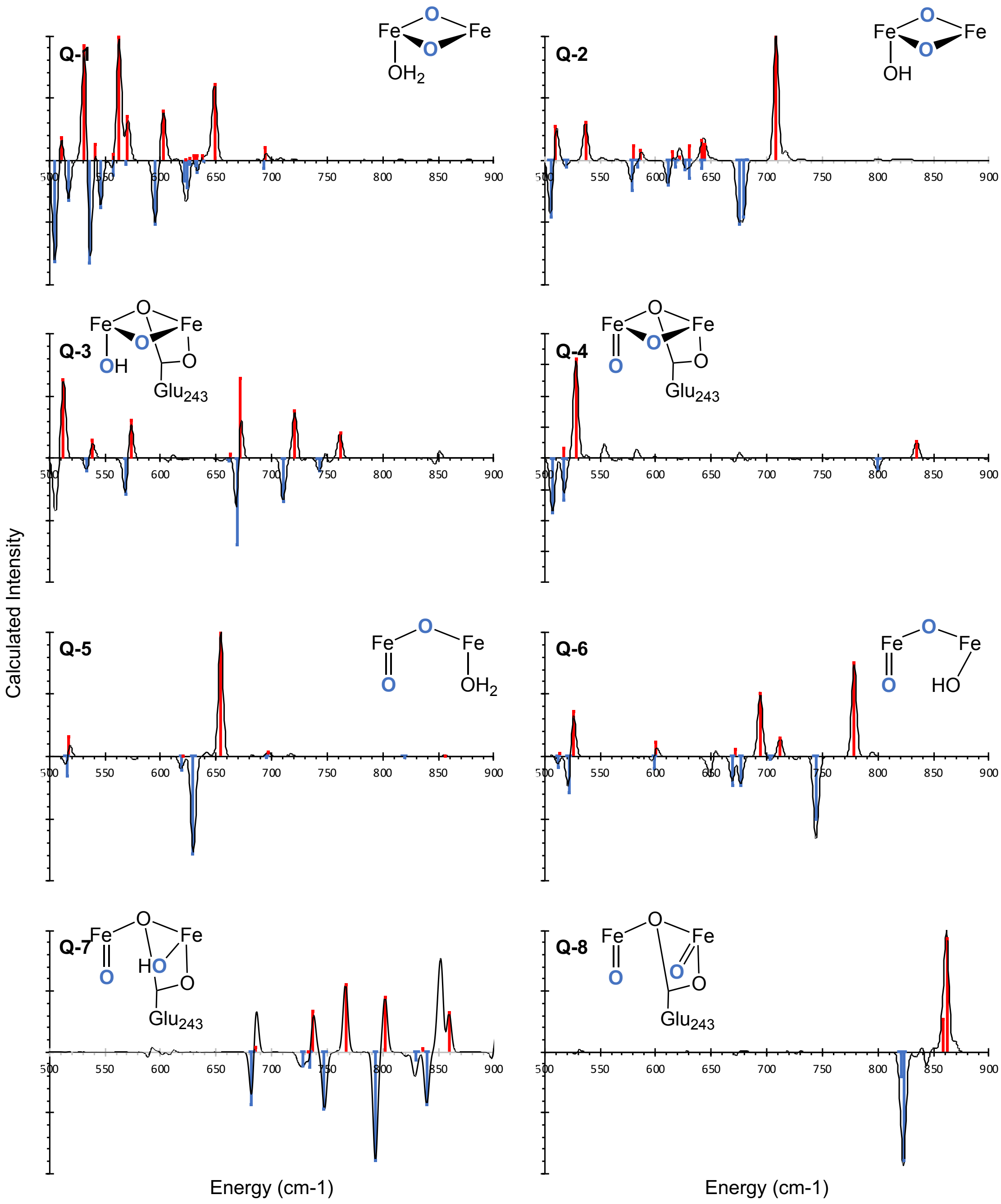

Figure S16. Non-resonant Raman ^{16}O-^{18}O difference spectra (black) with ^{16}O (red) and ^{18}O (blue decomposition from the full QM Hessian. Insets showing the structure and isotope labeled oxygens (blue).

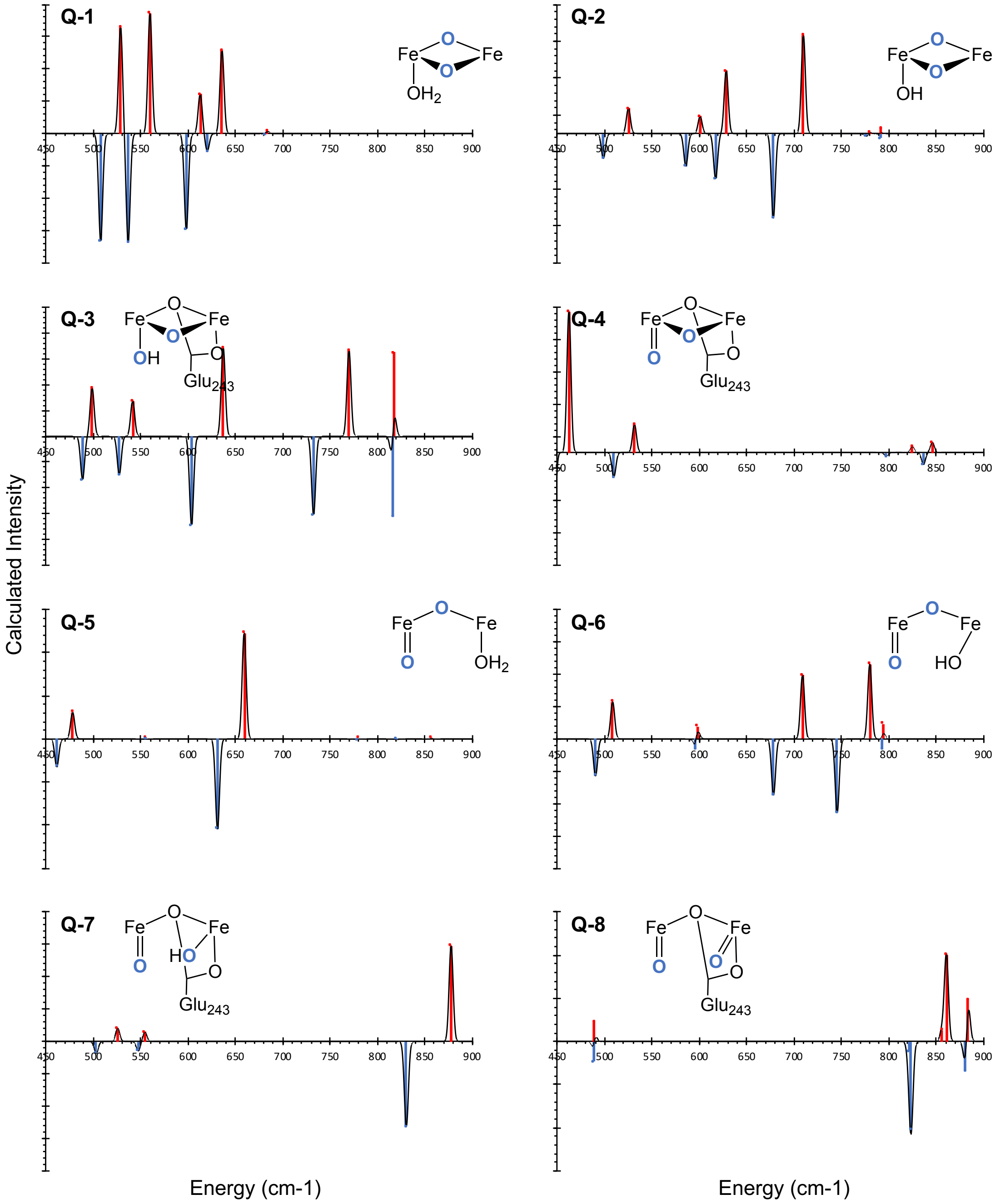

Figure S17. Non-resonant Raman ^{16}O-^{18}O difference spectra (black) with ^{16}O (red) and ^{18}O (blue decomposition from a partial Hessian only including the Fe_2O_x core. Insets showing the structure and isotope labeled oxygens (blue).

Table S7. Characteristic modes in the non-resonant Raman $^{16\text{-}18}O$ difference spectra and their respective shifts, sorted by the motif. Note that for model Q-6, where different labeling patterns were probed, multiple entries to the same mode with different shifts are obtained.

	Model	^{16}O	^{18}O	Shift
bis-μ-oxo	Q-1	531.57	517.19	-14.38
	Q-2	536.98	519.34	-17.64
	Q-1	562.73	545.99	-16.74
μ-oxo	Q-6	489.86	489.72	-0.14
	Q-6	489.86	479.57	-10.29
	Q-6	489.86	467.52	-22.34
	Q-3	538.84	533.76	-5.08
	Q-3	573.15	568.51	-4.64
	Q-5	654.26	629.47	-24.79
tOH	Q-6	598.95	583.65	-15.30
	Q-6	598.95	587.14	-11.81
	Q-6	598.95	590.39	-8.56
	Q-6	638.95	621.14	-17.81
	Q-6	638.95	634.14	-4.81
	Q-6	638.95	634.62	-4.33
	Q-2	641.56	630.16	-11.40
	Q-3	671.28	669.32	-1.96

	Q-7	685.84	682.25	-3.59
	Q-2	708.25	675.42	-32.83
	Q-3	720.65	710.28	-10.37
	Q-7	737.51	734.16	-3.35
	Q-7	766.39	747.55	-18.84
	Q-7	801.99	793.33	-8.66
tO	Q-6	764.65	760.74	-3.91
	Q-6	764.65	738.79	-25.86
	Q-6	764.65	736.93	-27.72
	Q-4	834.72	800	-34.72
	Q-7	850.3	828.57	-21.73
	Q-5	855.58	819.21	-36.37
	Q-7	859.55	839.49	-20.06
	Q-8	861.85	823.07	-38.78

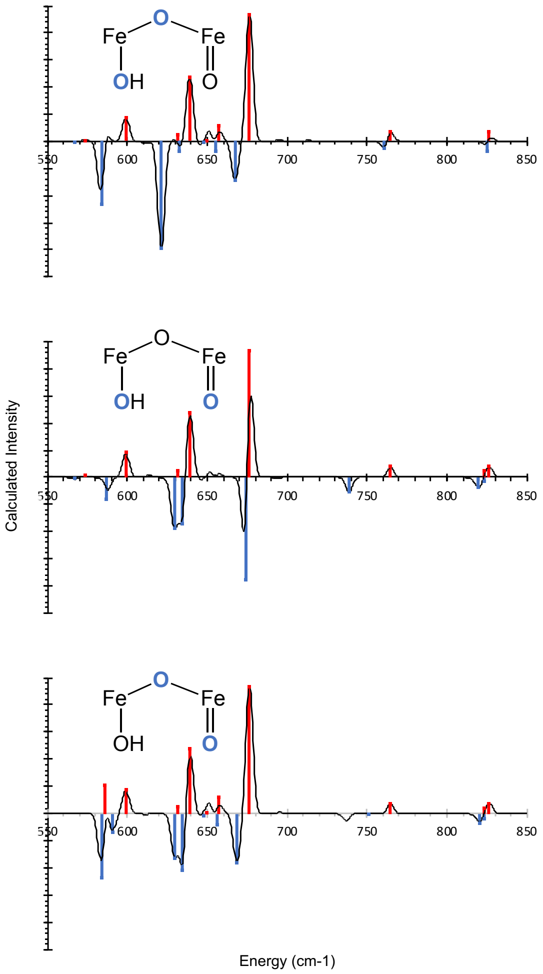

Figure S18. Non-resonant Raman ^{16}O-^{18}O difference spectra (black) with ^{16}O (red) and ^{18}O (blue decompos5tion for different labeling patterns in model Q6 (inlets).

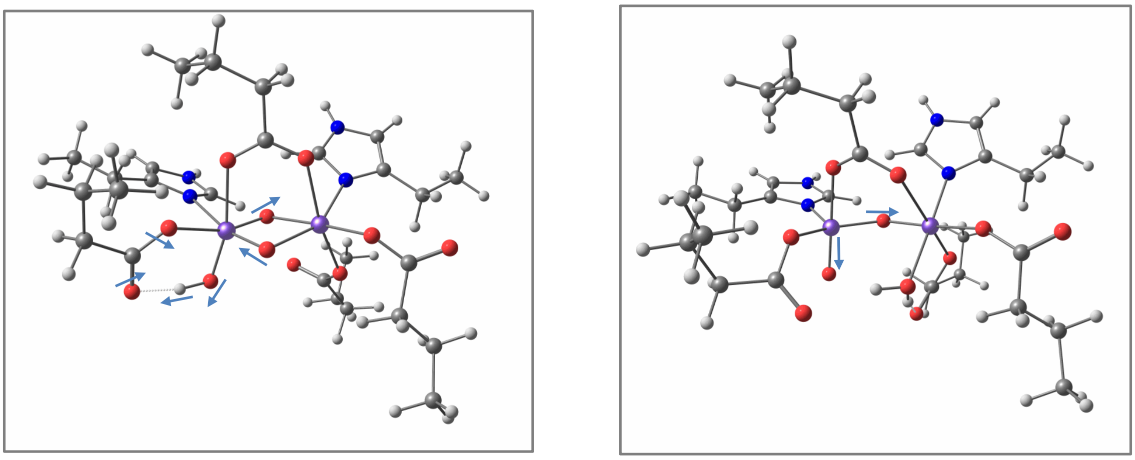

Figure S19. Schematic descriptoion of the relevant Raman modes of model **Q2** and **Q5**.

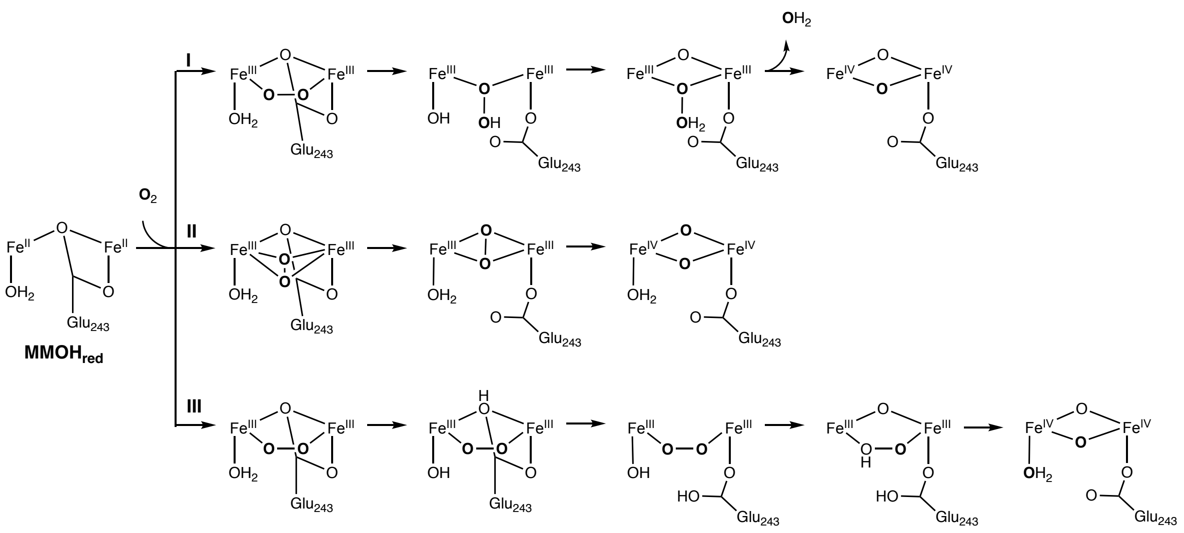

Scheme S1. Possible $MMOH_P$ to $MMOH_Q$ transitions, starting from $MMOH_{red}$ assuming a closed bis-μ-oxo core for $MMOH_Q$.[9] Oxygen atoms derived from molecular oxygen are labelled bold.

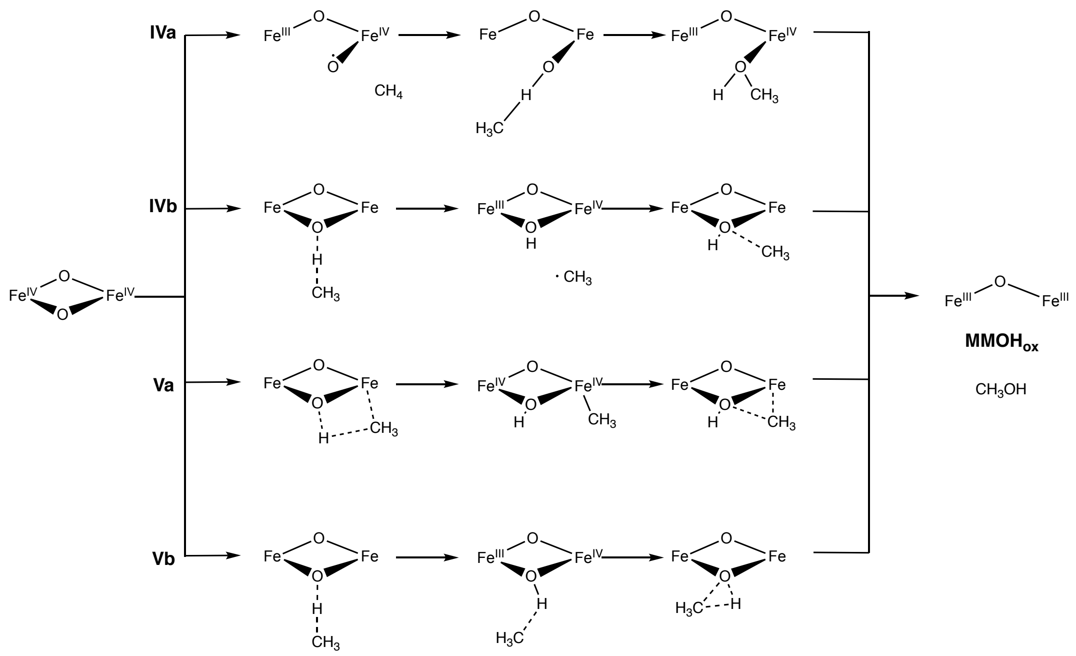

Scheme S2. Possible $MMOH_Q$ to $MMOH_{OX}$ transitions for bis-μ-oxo cores.[10]

References

(1) Whittington, D. A.; Lippard, S. J. Crystal structures of the soluble methane monooxygenase hydroxylase from Methylococcus capsulatus (Bath) demonstrating geometrical variability at the dinuclear iron active site. *J. Am. Chem. Soc.* **2001**, *123*, 827-838.

(2) Elango, N.; Radhakrishnan, R.; Froland, W. A.; Wallar, B. J.; Earhart, C. A.; Lipscomb, J. D.; Ohlendorf, D. H. Crystal structure of the hydroxylase component of methane monooxygenase from Methylosinus trichosporium OB3b. *Protein Sci.* **1997**, *6*, 556-568.

(3) Cutsail, G. E.; Banerjee, R.; Zhou, A.; Que, L.; Lipscomb, J. D.; DeBeer, S. High-Resolution Extended X-ray Absorption Fine Structure Analysis Provides Evidence for a Longer Fe···Fe Distance in the Q Intermediate of Methane Monooxygenase. *J. Am. Chem. Soc.* **2018**, *140*, 16807-16820.

(4) Jackson Rudd, D.; Sazinsky, M. H.; Merkx, M.; Lippard, S. J.; Hedman, B.; Hodgson, K. O. Determination by X-ray Absorption Spectroscopy of the Fe− Fe Separation in the Oxidized Form of the Hydroxylase of Methane Monooxygenase Alone and in the Presence of MMOD. *Inorg. Chem.* **2004**, *43*, 4579-4589.

(5) Rosenzweig, A. C.; Brandstetter, H.; Whittington, D. A.; Nordlund, P.; Lippard, S. J.; Frederick, C. A. Crystal structures of the methane monooxygenase hydroxylase from Methylococcus capsulatus (Bath): Implications for substrate gating and component interactions. *Proteins: Struct., Funct., Bioinf.* **1997**, *29*, 141-152.

(6) Whittington, D. A.; Sazinsky, M. H.; Lippard, S. J. X-ray Crystal Structure of Alcohol Products Bound at the Active Site of Soluble Methane Monooxygenase Hydroxylase. *J. Am. Chem. Soc.* **2001**, *123*, 1794-1795.

(7) Sazinsky, M. H.; Lippard, S. J. Product Bound Structures of the Soluble Methane Monooxygenase Hydroxylase from Methylococcus capsulatus (Bath): Protein Motion in the α-Subunit. *J. Am. Chem. Soc.* **2005**, *127*, 5814-5825.

(8) Castillo, R. G.; Banerjee, R.; Allpress, C. J.; Rohde, G. T.; Bill, E.; Que Jr, L.; Lipscomb, J. D.; DeBeer, S. High-Energy-Resolution Fluorescence-Detected X-ray Absorption of the Q Intermediate of Soluble Methane Monooxygenase. *J. Am. Chem. Soc.* **2017**, *139*, 18024-18033.

(9) Tinberg, C. E.; Lippard, S. J. Dioxygen Activation in Soluble Methane Monooxygenase. *Acc. Chem. Res.* **2011**, *44*, 280-288.

(10) Huang, S. P.; Shiota, Y.; Yoshizawa, K. DFT study of the mechanism for methane hydroxylation by soluble methane monooxygenase (sMMO): effects of oxidation state, spin state, and coordination number. *Dalton Trans.* **2013**, *42*, 1011-1023.
